# Comparative efficacy of Chinese herbal injections in patients with cardiogenic shock (CS): a systematic review and Bayesian network meta-analysis of randomized controlled trials

**DOI:** 10.3389/fphar.2024.1348360

**Published:** 2024-02-27

**Authors:** Linkai Yue, Lu Xiao, Xuemin Zhang, Liqing Niu, Yue Wen, Xiaowei Li, Ying Wang, Guanghe Xing, Guiwei Li

**Affiliations:** ^1^ Department of Emergency, First Teaching Hospital of Tianjin University of Traditional Chinese Medicine, Tianjin, China; ^2^ National Clinical Research Center for Chinese Medicine Acupuncture and Moxibustion, Tianjin, China

**Keywords:** network meta-analysis, cardiogenic shock, Chinese herbal injections, combination therapy, vasoactive medications

## Abstract

**Background:** Cardiogenic shock (CS) is the primary cause of death in patients suffering acute myocardial infarction. As an emerging and efficacious therapeutic approach, Chinese herbal injections (CHIs) are gaining significant popularity in China. However, the optimal CHIs for treating CS remain uncertain.

**Method:** We searched eight databases from inception to 30 September 2023. Subsequently, we conducted the Bayesian network meta-analysis (NMA). Interventions were ranked based on the surface under the cumulative ranking curve (SUCRA) probability values. To compare the effects of CHIs on two distinct outcomes, a clustering analysis was performed. Furthermore, the quality of the studies was assessed.

**Results:** For the study, we included 43 RCTs, encompassing 2,707 participants. The study evaluated six herbal injections, namely, Shenfu injection (SF), Shengmai injection (SM), Shenmai injection (Sm), Danshen injection (DS), Huangqi injection (HQ), and Xinmailong injection (XML). The analysis findings suggested that Sm (MD = −1.05, 95% CI: −2.10, −0.09) and SF (MD = −0.81, 95% CI: −1.40, −0.25) showed better efficacy compared to Western medicine (WM) alone in reducing in-hospital mortality. The SUCRA values revealed that Sm + WM ranked first in terms of in-hospital mortality, cardiac index (CI), and hourly urine output but second in improving left ventricular ejection fraction (LVEF) and mean arterial pressure (MAP). SF + WM, however, had the greatest impact on raising the clinical effective rate. In MAP, SM + WM came out on top. Moreover, in terms of safety, only 14 studies (31.8%), including five types of CHIs: SF, Sm, SM, HQ, and XML, observed adverse drug reactions.

**Conclusion:** To summarize, this analysis discovered that, in terms of patients suffering from CS, CHIs + WM yielded significantly greater advantages than WM alone. Based on in-hospital mortality and the remaining outcomes, Sm performed excellently among all the involved CHIs.

**Systematic Review Registration**: https:// www.Crd.york.ac.uk/prospero/, identifier: CRD42022347053.

## Introduction

Cardiogenic shock (CS) is a severe syndrome that is marked by end-organ hypoperfusion and hypotension as a result of ventricular pump failure and significantly reduced cardiac output caused by myocardial injury from various sources (van Diepen et al., 2017). CS affects over 40,000 Americans annually who have suffered acute myocardial infarction, with a mortality ranging from 40% to 50% (Samsky et al., 2021). In addition, patients who have combined CS have a worse prognosis than those who have acute myocardial infarction without it (Redfors et al., 2015). Therefore, it is critical to lower this high mortality, improve cardiac function, and increase tissue perfusion.

Vasoactive medications, inotropic drugs, early revascularization (ERV), mechanical circulatory support (MCS), and organ function support, such as mechanical ventilation, are now the major treatments for CS (Thiele et al., 2021). Vasoactive medications and inotropic drugs, which form the foundation of therapeutic interventions, are crucial for increasing cardiac output, enhancing tissue perfusion, and preserving hemodynamic stabilization. Nevertheless, this approach often comes with the trade-off of increased myocardial oxygen consumption, the potential for severe arrhythmias, and impaired cardiac and renal function (Tarvasmäki et al., 2016). Although the SHOCK trials (Hochman et al., 2006; 2001; 1999) show that ERV can effectively increase the long-term survival benefits of patients compared to drug treatment, there is no advantage of ERV in reducing 30-day mortality. Moreover, the ERV strategy needs to be comprehensively considered by the medical team, which poses a challenge for clinicians (Neumann et al., 2019). Additionally, MCS devices like the intra-aortic balloon pump (IABP), extracorporeal membrane oxygenation (ECMO), and percutaneous left ventricular assist devices (LVAD) have demonstrated promising results in decreasing myocardial oxygen consumption, raising cardiac output, lowering catecholamines, and stabilizing hemodynamics (Wong and Sin, 2020). However, based on the IABP-SHOCKII study (Thiele et al., 2013), IABP cannot lower mortality at 6 months and 12 months. The survival benefits of patients undergoing ECMO treatment have improved, and complications have significantly decreased (Schmidt et al., 2015; Hou et al., 2016; Ouweneel et al., 2016). However, considering its complex operation, high cost, and risk of increased myocardial oxygen consumption, the clinical application of ECMO is limited (Yang et al., 2014; Zhong et al., 2016). As for percutaneous LVAD, it can significantly improve hemodynamic parameters (Burkhoff et al., 2006; Seyfarth et al., 2008). However, in terms of improving the left ventricular ejection fraction (LVEF) and reducing mortality, percutaneous LVAD has not shown better efficacy than IABP (Ouweneel et al., 2017). Therefore, it is imperative that adjuvant therapy with excellent efficacy, a low risk of complications, and a low cost be used to improve the prognosis for CS.

Chinese herbal injections (CHIs) have gained significant popularity in the management of cardiovascular disorders, including the treatment of CS and other related conditions. By combining clinical experience in drug use with electronic database searches, we found that six herbal injections: Shenfu injection (SF), Shengmai injection (SM), Shenmai injection (Sm), Danshen injection (DS), Huangqi injection (HQ), and Xinmailong injection (XML) were used for the treatment of CS. They may enhance myocardial contractility, reduce myocardial oxygen consumption, and improve tissue hypoperfusion by enhancing antioxidant enzyme activity, preventing platelet aggregation, and regulating the expression of inflammatory factors (Yang and Wang, 2021). However, no trials have been conducted comparing the CHIs mentioned directly. The efficacy and safety of individual herbal injections, which are assessed by only a few systematic reviews (Yang et al., 2012; Zhang et al., 2015), provide little room for ranking them. Therefore, it is unclear which CHI has the best efficacy against CS. In contrast, network meta-analysis (NMA), which combines direct comparative evidence with indirect comparative evidence, is able to rank the effects of multiple interventions. In order to assist medical professionals in selecting the best treatment in clinical practice, we ranked the effectiveness and discussed the safety of the aforementioned CHIs plus Western medicine (WM) using NMA within the Bayesian net framework.

## Materials and methods

### Standard evaluation of Chinese herbal injection

To ensure the accuracy of the study, this analysis adopted the ConPhyMP consensus (Heinrich et al., 2022) as a reference when reporting CHIs. Simultaneously, we followed the guidelines (Rivera et al., 2014) for standardizing the scientific nomenclature of botanical drug components. Moreover, we validated these names by cross-referencing them with the websites of “Plant of the World Online” (http://www.plantsoftheworldonline.org) and “The World Flora Online” (WFO, http://www.worldfloraonline.org/). Based on the principles outlined in the four pillars of ethnopharmacology, summary tables were prepared to describe the composition of agents and their reporting in the original study. The composition of the included CHIs is displayed in Supplementary Table S1. Further details are depicted in Supplementary Tables S2–S4 (Supplementary S2, S3).

### Study registration

We performed this systematic review in accordance with the Preferred Reporting Items for Systematic Reviews and Network Meta-Analysis (PRISMA-NMA) statement (Supplementary S4) (Hutton et al., 2015). And the protocol had been registered prospectively under the registration number CRD42022347053.

### Inclusion and exclusion criteria

This review encompassed five inclusion criteria: 1. Age: >18 years; 2. Study design: randomized controlled trials (RCTs); 3. Diagnostic criteria: a diagnosis of CS in accordance with the “Acute Myocardial Infarction Guidelines for Diagnosis and Treatment” (Chinese Society of Cardiology et al., 2001) and the “Contemporary Management of Cardiogenic Shock: A Scientific Statement From the American Heart Association” (van Diepen et al., 2017); 4. Treatments: all patients received WM, including mechanical ventilation, ERV, vasoactive medications, MCS, and nutritional support. Based on the aforementioned treatments, the experimental group was administered a specific type of CHIs, while the control group received a second, or WM only. There was no restriction on the duration. 5. Outcomes: in-hospital mortality was considered the primary outcome since it served as a vitally important indicator of the overall effectiveness of interventions and the survival benefits they provided to patients in such a life-threatening condition. And eight outcomes, including cardiac function indicators: the cardiac index (CI) and the LVEF, the mean arterial pressure (MAP), the peripheral tissue perfusion indicator: the hourly urine output, the myocardial injury indicator: the level of cardiac troponin I (cTnI), the inflammation indicator: the level of c-reactive protein (CRP), the clinical effective rate, and the safety indicator: adverse drug reactions (ADRs)/adverse drug events (ADEs), were regarded as secondary outcomes. The calculation formula employed to determine in-hospital mortality was the number of deceased patients divided by the number of patients during hospitalization. The formula applied for calculating the clinical effective rate was (number of cured patients + number of improved patients)/number of all the patients. If the symptoms and/or signs of shock in the patient resolved and the patient returned to a normal state, it was considered cured. Patients were deemed effective if their shock symptoms and/or signs improved. If there was no improvement in the patient’s shock symptoms or signs, or if there was even a deterioration in the condition or the patient’s death, it was judged to be ineffective. If the study met the following criteria, it was considered unqualified: 1) Combined with other types of shock, such as septic shock, neurogenic shock, allergic shock, hemorrhagic shock, etc., 2) The study included specific populations: patients with severe lung, liver, kidney, hematopoietic, and cerebrovascular diseases. 3) The complete text of the literature could not be obtained, or only a summary. 4) Incomplete, incorrect, or unavailable literature data.

### Search strategy

Literature published before 30 September 2023, in electronic databases such as PubMed, Embase, The Cochrane Library, Web of Science, the China National Knowledge Infrastructure (CNKI), the China Biology Medicine disc (CBM), the Wanfang Database, and the Chinese Scientific Journal database (VIP) was searched comprehensively. Medical subject headings (MeSH) and free words were employed to retrieve literature. There was no language restriction in the NMA. In addition, we conducted manual searches of relevant meta-analyses, systematic reviews, and references to related studies. In Supplementary S5, the search strategy’s complete details are displayed.

### Literature selection and data extraction

A thorough evaluation of all the literature was conducted by two researchers (LK Yue and L Xiao), working independently and in parallel. To ensure accuracy, all duplications in the literature were removed in the initial stage. Subsequently, a meticulous examination of the titles and abstracts allowed the researchers to eliminate reviews and studies that were not pertinent to the research question. Then, the researchers screened the studies that fulfilled the predetermined inclusion criteria by thoroughly examining the complete texts. Any inconsistencies in the literature screening process were resolved through full discussion or by the arbitrator (GW Li). Microsoft Excel 2021 was used to create a spreadsheet to extract and enter information.

### Risk of bias assessment

In order to analyze the bias in each RCT, we used the revised Cochrane risk-of-bias tool for randomized trials (RoB2) (Sterne et al., 2019). It contains bias across the following domains: the randomization process, deviations from intended interventions, missing outcome data, measurement of outcomes, and selective reporting. Some signal problems are set up in each domain. Considering all the information comprehensively, RoB2 divided the overall bias risk into three grades: “high,” “some concerns,” and “low.” Two independent evaluators (LK Yue and L Xiao) conducted the entire bias risk assessment process. When their opinions were inconsistent, the consensus was reached or solved by a third researcher (GW Li).

The evaluation of the certainty of the evidence obtained from this NMA was conducted utilizing the Confidence in Network Meta-Analysis (CINeMA) application, providing the opportunity to assess the levels of confidence (Nikolakopoulou et al., 2020).

### Statistical analysis

We used Stata 17.0 and R 4.2.3 for calculation and drawing. By employing the BUGSnet packages, gemtc packages, and rjags packages, statistical analysis was conducted through the utilization of Markov chain Monte Carlo (MCMC) simulation techniques. For continuous variables, we used the 95% confidence intervals (CIs) of the mean differences (MDs) as the combined result. We conducted an analysis of the binary variables by calculating the odds ratios (ORs) and their corresponding 95% confidence intervals (CIs). Statistical significance was inferred when the 95% CIs of the mean differences (MDs) did not encompass 0 ORs and when the 95% CIs of the ORs did not encompass 1. Firstly, we fitted both the random and fixed effect models. Simulation analysis employed 4 chains with 20,000 annealing times, a step size of 1, and 50,000 simulation iteration times as the parameters. The model that had a lower deviation information criterion (DIC) value indicated a higher fitting degree (Béliveau et al., 2019). In the case of a closed loop, we examined the overall network consistency by conducting the unrelated mean effect (UME) model (Veroniki et al., 2014; Efthimiou et al., 2016). As for consistency, we employed the node-splitting method to evaluate it (van Valkenhoef et al., 2016). However, all outcomes in this NMA were non-closed loops, the consistency hypothesis was not applicable in this study. The CHIs were ranked using the calculated surface under the cumulative ranking curve (SUCRA) values (Salanti et al., 2011; Riley et al., 2017). At last, funnel plots were performed to analyze publication bias (Chaimani et al., 2013).

## Results

### Literature selection

**FIGURE 1:**
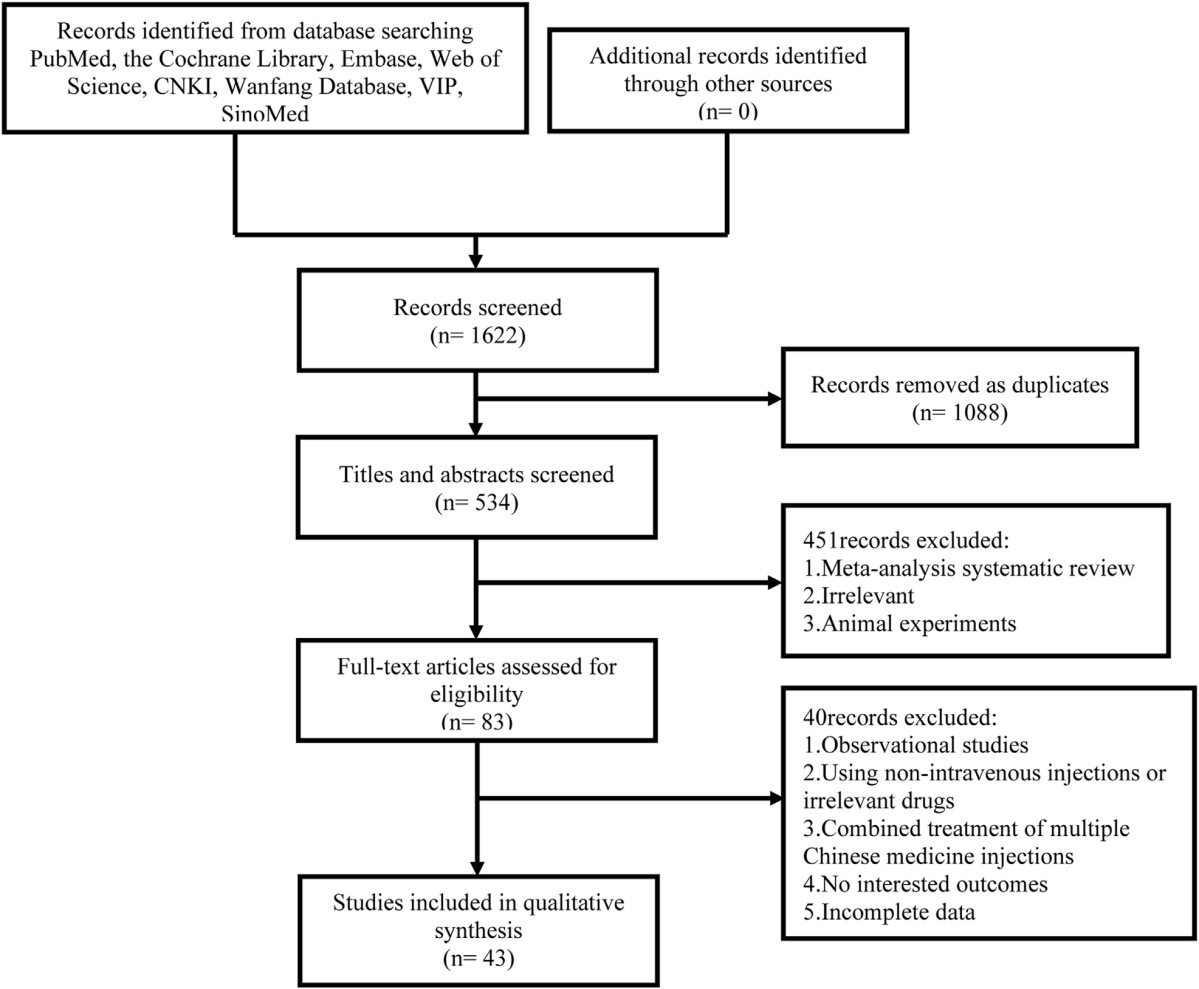
Flow diagram of study inclusion.

We obtained 1,622 studies in the initial search. Among them, 1,088 studies were identified as duplicates and were excluded. By reviewing the titles and abstracts, we eliminated 451 studies due to their inclusion of reviews, research unrelated to the topic, or animal experiments. After that, we evaluated the remaining 83 related studies by reading the full text. Finally, 43 studies were incorporated into the analysis, involving six different CHIs: SF, SM, Sm, DS, HQ, and XML. 40 RCTs were excluded in accordance with the following criteria: 1. Observational studies (n = 11); 2. Using unrelated medicine (n = 14); 3. Absence of diagnostic criteria for the disease or incomplete reporting of outcomes (n = 7); 4. Incomplete data (n = 2); 5. Combined use of multiple CHIs (n = 6). Figure 1 displays a visual representation of the literature selection.

### Study characteristics

**FIGURE 2:**
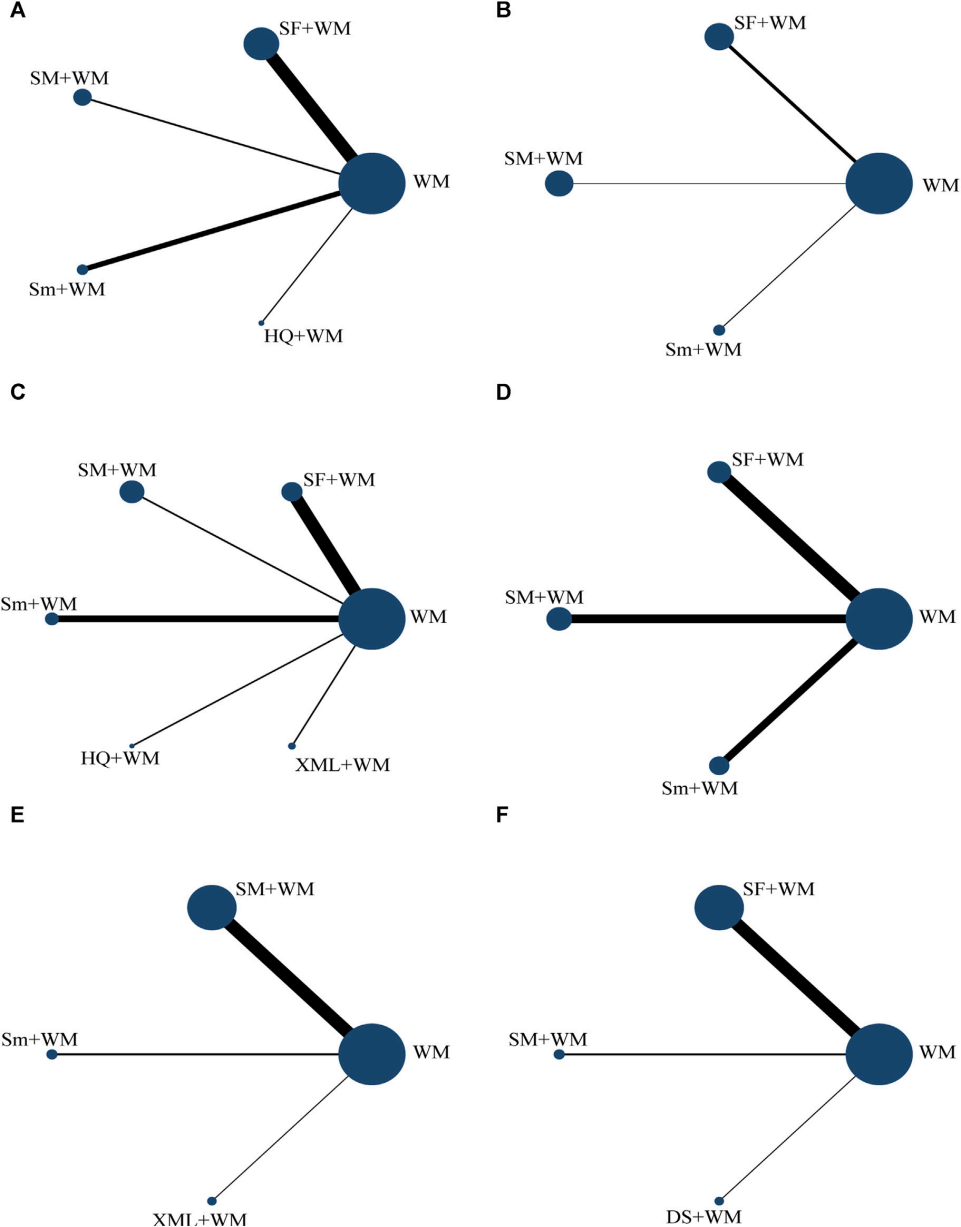
Network graph of the different outcomes. The lines’ width represents the number of studies on two CHIs for direct comparison. The size of the spot area signifies the number of individuals involved in the research. **(A)** In-hospital mortality; **(B)** cardiac index (CI); **(C)** left ventricular ejection fraction (LVEF); **(D)** mean arterial pressure (MAP); **(E)** hourly urine output; **(F)** clinical effective rate.

Under the Bayesian framework, we included 43 RCTs that were carried out in China between 2000 and 2022. The NMA included a diverse sample size, ranging from 20 individuals to 196 individuals, with 2,707 participants. Six types of CHIs were encompassed, like SF (n = 21), SM (n = 12), Sm (n = 7), DS (n = 1), HQ (n = 1), and XML (n = 1). The control group was treated with mechanical ventilation, vasoactive drugs, MCS, or nutritional support. Vasoactive drugs mainly contain norepinephrine, epinephrine, isoproterenol, dopamine, dobutamine, metaraminol, milrinone, and Levosimendan. The intervention group received one kind of the aforementioned herbal injections as a therapeutic intervention, in contrast to the control group. No limitation existed in the duration of therapy. Table 1 (Supplementary S6) and Figure 2 depict more details.

**TABLE 1:** Characteristics of the studies included in the network meta-analysis.

Study ID	N (E/C)	Sex (M/F)	Age (years)	Therapy of experiment group	Therapy of control group	Course (day)	Outcomes
Bi and Liu. (2005)	14/12	16/10	62.4 ± 10.2	Shenfu 40 mL Qd + WM	WM	14	③
Ding and Xu (2006)	15/15	16/14	57.8 ± 8.4	Shengmai 30 mL Q12h + WM	WM	7	①②⑤
Du (2018)	25/25	31/19	E:40.21 ± 7.77	Shenfu 50 mL Bid + WM	WM	1	②
			C:41.33 ± 7.56				
Fan and He (2013)	60/58	76/44	E: 69.3 ± 12.8	Shenfu 40 mL Qd + WM	WM	14	①③⑨
			C:70.1 ± 9.3				
Feng et al. (2016)	45/45	67/23	43.4 ± 7.1	Shengmai 60 mL Q12h + WM	WM	7	②④
Gao and Wang (2013)	28/18	27/19	E:65.3 ± 18.4	Shenfu 40 mL Qd + WM	WM	1	⑨
			C:65.1 ± 17.7				
Ge et al. (2018)	50/50	74/26	E:71.2 ± 11.5	Xinmailong 100–400 mg Bid + WM	WM	10	③⑤⑨
			C:70.8 ± 10.8				
Jiang (2017)	30/30	39/21	E: 70.71 ± 6.00	Shengmai 20 mL Qd + WM	WM	7	⑦
			C:69.43 ± 8.25				
Jin et al. (2016)	30/30	52/8	E:57 ± 12	ShenFu 100 mL Qd + WM	WM	3	①③⑦⑨
			C:60 ± 16				
Lan et al. (2014)	56/56	57/55	E:8.4 ± 11.7	Shengmai 60 mL Qd + WM	WM	7	②④⑤⑥
			C:59.6 ± 10.8				
Li (2007)	32/31	41/22	E:53.2 ± 10.7	Shengmai 30 mL Qd + WM	WM	7–10	⑥⑨
			C:51 ± 9.8				
Li (2016)	32/32	40/24	62.73 ± 8.23	Shenfu 100 mL Qd + WM	WM	14	①
Li et al. (2016)	40/40	40/40	62.5 ± 5.4	Shenmai 100 mL Qd + WM	WM	5	④⑤
Li and Li (2012)	18/18	19/17	E:56.3	Shengmai 60 mL Qd + WM	WM	7	⑥
			C:57.2				
Lin et al. (2020)	36/36	40/32	E:67.25 ± 13.28	Shenfu 80 mL Qd + WM	WM	7	①②③④⑦
			C:.85 ± 11.36				
Liu (2015)	30/30	33/27	E:44.2	Shengmai 100 mL Qd + WM	WM	1	③
			C:44.8				
Liu (2020)	10/10	13/7	E:5 ± 2.6	Shenfu 100 mL Qd + WM	WM	-	④⑨
			C:0.1 ± 2.4				
Liu (2007)	17/17	19/15	57.4 ± 17.2	Shenmai 50 mL Qd + WM	WM	14	①③⑨
Long et al. (2000)	15/15	22/8	68.5 ± 8.2	Shenmai 40 mL Qd + WM	WM	14	①③⑨
Mi et al. (2009)	30/29	36/23	E:62.33 ± 10.27	Huangqi 50 mL Qd + WM	WM	14	①③⑨
			C:60.43 ± 11.55				
Pan et al. (2015)	30/30	31/29	-	Shenfu 50 mL Bid + WM	WM	1	②⑥
Ren (2016)	20/20	19/21	E:61 ± 1.2	Shenfu 50 mL Qd + WM	WM	2	⑤
			C:59 ± 3.9				
Shi et al. (2018)	56/56	87/25	E:61.6 ± 7.2	Shenfu 200 mL Qd + WM	WM	5	②③④⑦⑨
			C:60.6 ± 5.0				
Shi and Liu (2011)	18/18	23/13	E:71	Shenfu 80 mL Qd + WM	WM	7	①③⑥⑨
			C:70				
Song (2018)	60/60	80/40	E:54.1 ± 4.6	Shenfu 40 mL Qd + WM	WM	7	②③⑥⑦⑨
			C:54.8 ± 4.2				
Song et al. (2022)	30/30	31/29	E:74.91 ± 11.50	Shenfu 100 mL Qd + WM	WM	7	②④
			C:77.22 ± 10.84				
Su et al. (2020)	30/30	42/18	E:63.87 ± 10.18	Shenfu 60 mL Qd + WM	WM	1	②④
			C:64.33 ± 9.70				
Su and Huang (2021)	40/40	43/37	E:64.75 ± 13.0	Shengmai 25–50 mL Qd + WM	WM	-	⑥
			C:64.19 ± 15.4				
Wang (2003)	20/22	31/11	E:59.40 ± 16.27	Shengmai 30–100 mL Qd + WM	WM	7	①⑥
			C:58.11 ± 17.27				
Wang and Zhang (2011)	25/25	35/15	46.5 ± 7.2	Shenmai 100 mL Q12h + WM	WM	7	④⑤⑥
Wei (2013)	26/26	25/27	64.1 ± 2.2	Shengmai 100 mL Bid + WM	WM	7	④⑤⑥
Wu and Teng (2001)	34/34	30/38	65	Shenmai 80 mL Q Qd + WM	WM	7	①⑨
Xiong (2009)	19/19	22/16	58.3 ± 17.9	Shenfu 60 mL Qd + WM	WM	14	①③
Xu et al. (2014)	32/32	36/28	E:72.23 ± 15.12	Shenfu 100 mL Qd + WM	WM	7–10	①②⑥
			C:71.47 ± 16.28				
Yang and Cai (2016)	98/98	101/95	E:57.03 ± 6.74	Shengmai 20–60 mL Qd + WM	WM	7	①③⑤⑥⑦
			C:56.27 ± 40.31				
Yu andLiu (2004)	32/30	39/23	E:62.0 ± 8.6	Shenmai 20–50 mL Qd + WM	WM	-	⑨
			C:64.0 ± 5.2				
Zhang et al. (2019)	32/32	35/29	E:69.4 ± 9.2	Shenfu 40 mL Qd + WM	WM	14	⑨
			C:68.5 ± 9.0				
Zhang et al. (2017)	30/28	34/24	49.21 ± 23.89	Danshen 20 mL Qd + WM	WM	14	⑥
Zhang et al. (2014)	13/12	16/9	51.2 ± 9.1	Shenmai 240–480 mL Qd + WM	WM	1–2	②③④
Zhang (2012)	32/32	42/22	47.6 ± 7.9	Shengmai 100 mL Bid + WM	WM	7	④⑤⑥
Zhang (2017)	23/23	27/19	65.4 ± 11.3	Shenfu 100 mL Qd + WM	WM	3	④
Zhao (2009)	13/13	15/11	E:59 ± 2	Shenfu 30 mL Qd + WM	WM	10–15	③⑥
			C:58 ± 4				
Zhao et al. (2017)	37/37	55/19	E:6.31 ± 4.24	Shenfu 50 mL Bid + WM	WM	3	⑧
			C:7.29 ± 4.27				

Note: ① In-hospital mortality; ② Cardiac index (CI); ③ Left ventricular ejection fraction (LVEF); ④ Mean arterial pressure (MAP); ⑤ Hourly urine output; ⑥ Clinical effective rate; ⑦ Level of cardiac troponin I (cTnI); ⑧ Level of c-reactive protein (CRP); ⑨ Adverse drug reactions (ADRs)/adverse drug events (ADEs). Abbreviations: Qd, quaque die (once a day); Bid, bis in die (twice a day); Q12h, quaque duodecim horas (every 12 h).

### Risk of bias assessment

**FIGURE 3:**
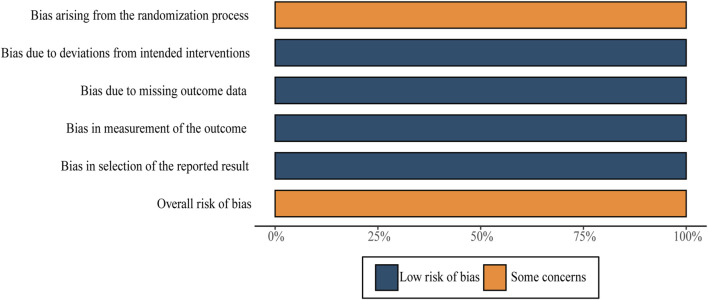
Risk-of-bias summary.

We applied RoB2 to this assessment. All RCTs in this evaluation were regarded as having “some concerns” due to undisclosed allocation concealment during the process of randomization. Every single RCT was classified as having “low risk of bias” in three domains: deviations from intended interventions, missing outcomes, and outcome measurements. Overall, the sum total of RCTs underwent assessment with “some concerns.” Further details are shown in Figure 3. Moreover, despite the lack of detailed information regarding blinding in most of the studies, it is improbable that the absence of blinding would have a substantial impact on the results.

### Certainty of the evidence

The levels of evidence from this NMA for primary and secondary outcomes were evaluated using the CINeMA tool. The confidence in estimates for all outcomes was deemed to be very low based on the CINeMA assessment. The detailed results are displayed in Supplementary S7.

### Model selection

The model fitting results indicated that there were four outcomes: in-hospital mortality (the model with random effects: DIC: 41.43, I^2^: 0%; the model with fixed effects: DIC: 39.56, I^2^: 0%); MAP (the model with random effects: DIC: 34.97, I^2^: 2%; the model with fixed effects: DIC: 33.43, I^2^: 7%); hourly urine output (the model with random effects: DIC: 18.99, I^2^: 0%; the model with fixed effects: DIC: 17.06, I^2^: 27%); and clinical effective rate (the model with random effects: DIC: 49.46, I^2^: 0%; the model with fixed effects: DIC: 48.11, I^2^: 3%) showed a higher level of fitting degree under the fixed effects model. However, for two other outcomes, namely, CI (the random effect model: DIC: 37.34, I^2^: 0%; the fixed effect model: DIC: 40.09, I^2^: 20%) and LVEF (the random effect model: DIC: 46.04, I^2^: 0%; the fixed effect model: DIC: 51.23, I^2^: 27%), the random effects model was employed. The I^2^ values of the best-fitting model of all outcomes were less than 50%, suggesting that the heterogeneity was acceptable. Because of non-closed rings, the consistency hypothesis was not established. Therefore, the UME model and the node-splitting method were also not applicable to this NMA.

## Outcomes

### In-hospital mortality

**FIGURE 4:**
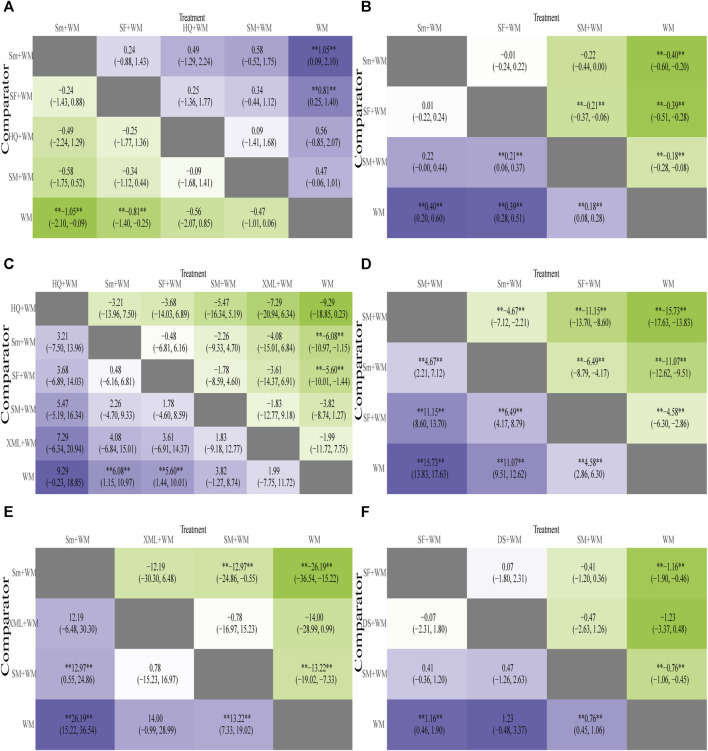
Heatmap of six outcomes. Mean difference (MD) with 95% confidence intervals (CIs) of the area under the receiver operating characteristic (AUC) of five outcomes of cardiogenic shock (CS). The values in each cell show how the risk score at the top compares to the risk score on the left in terms of relative impact. **Represents a statistically significant result. **(A)** In-hospital mortality; **(B)** cardiac index (CI); **(C)** left ventricular ejection fraction (LVEF); **(D)** mean arterial pressure (MAP); **(E)** hourly urine output; **(F)** clinical effective rate.

In-hospital mortality, the primary outcome, was recorded in 13 RCTs (Long et al., 2000; Wu and Teng, 2001; Ding and Xu, 2006; Liu, 2007; Mi et al., 2009; Xiong, 2009; Shi and Liu, 2011; Fan and He, 2013; Xu et al., 2014; Jin et al., 2016; Li, 2016; Yang and Cai, 2016; Lin et al., 2020). According to Figure 4A, when it came to reducing the in-hospital mortality, the combined approach of Sm + WM (MD = −1.05, 95% CI: −2.10, −0.09) and SF + WM (MD = −0.81, 95% CI: −1.40, −0.25) demonstrated a more pronounced impact compared to WM. However, the comparison of other interventions did not yield meaningful results.

**FIGURE 5:**
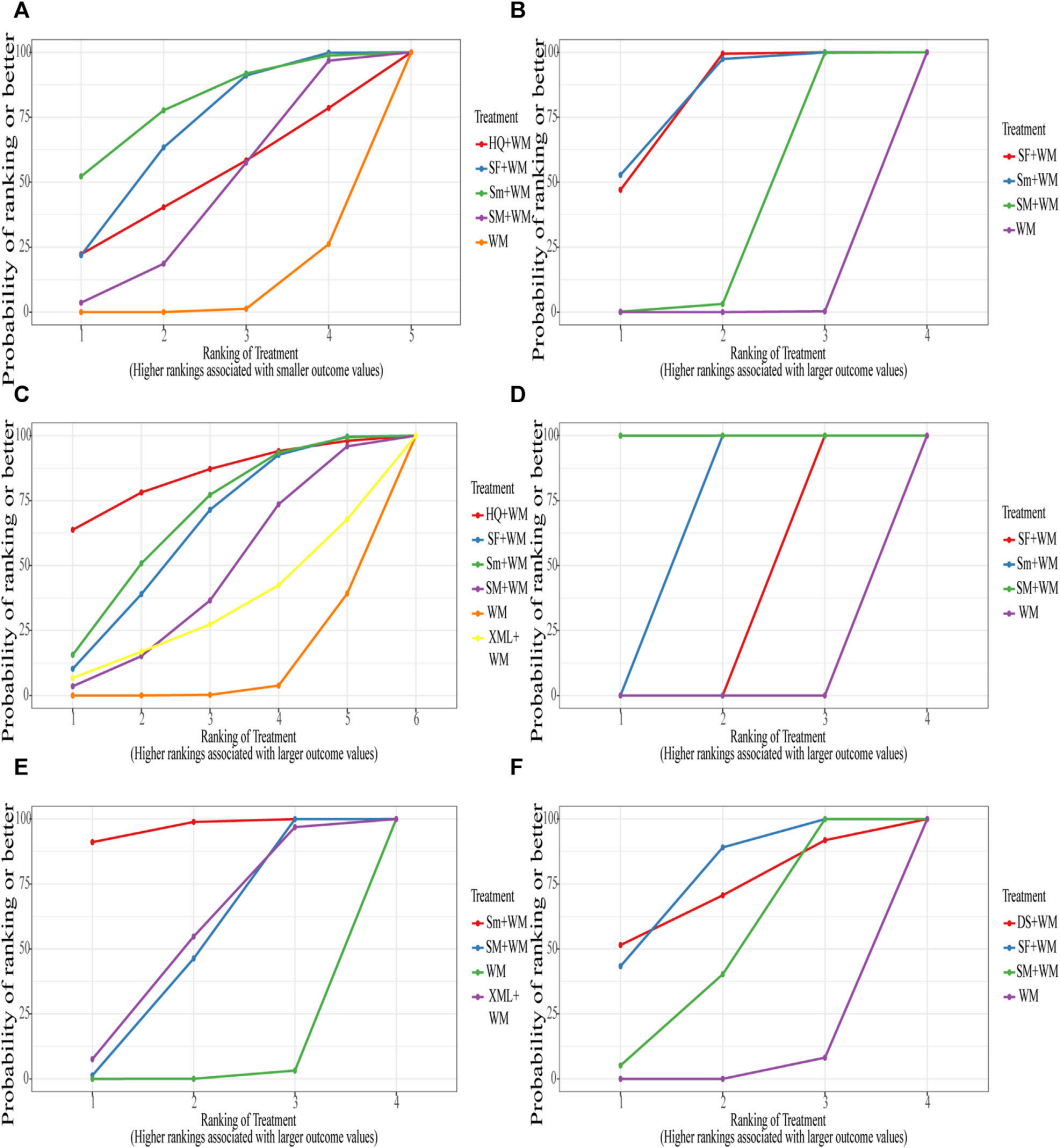
Surface under the cumulative ranking curve (SUCRA) Plot. **(A)** In-hospital mortality; **(B)** cardiac index (CI); **(C)** left ventricular ejection fraction (LVEF); **(D)** mean arterial pressure (MAP); **(E)** hourly urine output; **(F)** clinical effective rate.

Based on the SUCRA values displayed in Supplementary Table S14 (Supplementary S8) and Figure 5A, we ranked the interventions in the following order: Sm + WM, SF + WM, HQ + WM, SM + WM, and WM.

### Cardiac index

A total of 12 studies (Ding and Xu, 2006; Lan et al., 2014; Xu et al., 2014; Zhang et al., 2014; Pan et al., 2015; Feng et al., 2016; Song, 2018; Du, 2018; Shi et al., 2018; Lin et al., 2020; Su et al., 2020; Song et al., 2022) monitored CI, involving 3 types of CHIs. The results of the NMA were displayed in Figure 4B. Compared with WM, Sm combined with WM (Sm + WM: MD = 0.40, 95% CI: 0.20, 0.60), SF combined with WM (SF + WM:MD = 0.39, 95% CI: 0.28, 0.51), and SM combined with WM (SM + WM:MD = 0.18, 95% CI: 0.08, 0.28) performed better in improving CI. In addition, based on WM, SF was superior to SM in enhancing CI (SF + WM vs. SM + WM: MD = 0.21, 95% CI: 0.06, 0.37). However, no substantial disparity was observed among the remaining CHIs.

The ranking of SUCRA values also strongly proved this result: Sm + WM, SF + WM, SM + WM, and WM in Table 2 (Supplementary S8) and Figure 5B.

### Left ventricular ejection fraction

LVEF was assessed in 15 studies (Long et al., 2000; Bi and Liu, 2005; Liu, 2007; Mi et al., 2009; Xiong, 2009; Zhao, 2009; Shi and Liu, 2011; Fan and He, 2013; Zhang et al., 2014; Liu, 2015; Yang and Cai, 2016; Ge et al., 2018; Shi et al., 2018; Song, 2018; Lin et al., 2020). The results of NMA are depicted in Figure 4C. In terms of improving LVEF, Sm + WM (MD = 6.08, 95% CI: 1.15, 10.97) and SF + WM (MD = 5.60, 95% CI: 1.44, 10.01) outperformed WM alone.

According to the SUCRA values shown in Table 2 (Supplementary S8) and Figure 5C, the order was: HQ + WM, Sm + WM, SF + WM, SM + WM, XML + WM, WM.

### Mean arterial pressure

12 studies (Wang and Zhang, 2011; Zhang, 2012; Wei, 2013; Lan et al., 2014; Zhang et al., 2014; Li et al., 2016; Zhang et al., 2017; Shi et al., 2018; Lin et al., 2020; Liu, 2020; Su et al., 2020; Song et al., 2022) reported MAP in patients. The detailed results are shown in Figure 4D. SM + WM (MD = 15.73, 95% CI: 13.83, 17.63), Sm + WM (MD = 11.06, 95% CI: 9.51, 12.62), and SF + WM (MD = 4.58, 95% CI: 2.86, 6.30) improved MAP more effectively than WM alone. On the basis of WM, SM noticeably performed better than Sm and SF (SM + WM vs. Sm + WM: MD = 4.67, 95% CI: 2.21, 7.12; SM + WM vs. SF + WM: MD = 11.15, 95% CI: 8.60, 13.70).

SUCRA values further supported the above results. SM + WM had the best curative effect, followed by Sm + WM and SF + WM. The specific results were shown in Table 2 (Supplementary S8) and Figure 5D.

### Hourly urine output

Hourly urine output was estimated in 10 RCTs (Ding and Xu, 2006; Wang and Zhang, 2011; Zhang, 2012; Wei, 2013; Lan et al., 2014; Feng et al., 2016; Li et al., 2016; Ren, 2016; Yang and Cai, 2016; Ge et al., 2018) with 3 interventions. Details were displayed in Figure 4E. Sm + WM (MD = 26.19, 95% CI: 15.22, 36.54) and SM + WM (MD = 13.22, 95% CI: 7.33, 19.02) had a more outstanding performance than WM in increasing hourly urine output. Furthermore, based on WM, Sm had an obviously higher hourly urine output than SM. However, there was no significant difference between XML combined with WM (XML + WM: MD = 14.00, 95% CI: −0.99, 28.99) and WM. The differences in the comparisons of the other CHIs suggested no statistical significance.

The order depended on SUCRA values: Sm + WM, XML + WM, SM + WM. Details of the results were recorded in Table 2 (Supplementary S8) and Figure 5E.

### The clinical effective rate

We found that this outcome was evaluated in 15 RCTs (Wang, 2003; Li, 2007; Zhao, 2009; Shi and Liu, 2011; Wang and Zhang, 2011; Li and Li, 2012; Zhang, 2012; Wei, 2013; Lan et al., 2014; Xu et al., 2014; Pan et al., 2015; Yang and Cai, 2016; Zhang, 2017; Song, 2018; Su and Huang, 2021). The results are shown in Figure 4F. Compared with WM, SF + WM (MD = 1.16, 95% CI: 0.46, 1.90) and SM + WM (MD = 0.76, 95% CI: 0.45, 1.06) showed a more evident impact on the clinical effective rate. The efficacy of DS plus WM (DS + WM: MD = 1.23, 95% CI: −0.48, 3.37) compared to WM or the rest of the treatments displayed little significant difference.

The order of SUCRA values was also such that SF + WM performed more excellently than SM + WM. More information is illustrated in Table 2 (Supplementary S8) and Figure 5F.

### The level of cTnI

Six studies (Song, 2018; Shi et al., 2018; Yang and Cai, 2016; Lin et al., 2020; Jiang, 2017; Jin et al., 2016) reported cTnI. Three studies involved SF, 2 studies involved SM, and 1 study involved Sm. The results from the aforementioned six studies exhibited statistical significance.

### The level of CRP

Only one of the included studies (Zhao et al., 2017) recorded CRP. In this study, SF + WM had a better effect on reducing CRP levels than WM.

### ADRs/ADEs

A total of 14 RCTs (Long et al., 2000; Wu and Teng, 2001; Wang, 2003; Ding and Xu, 2006; Liu, 2007; Mi et al., 2009; Xiong, 2009; Shi and Liu, 2011; Fan and He, 2013; Xu et al., 2014; Jin et al., 2016; Li, 2016; Yang and Cai, 2016; Lin et al., 2020) that observed ADRs or ADEs among the 42 studies were included. One RCT (Shi et al., 2018) reporting ADRs of SF documented 1 case of gingival bleeding, 2 cases of palpitations, 2 cases of skin itching, and 1 case of dizziness. In another RCT (Zhang and Hu, 2019), there was documentation of a case of dizziness, a case of abdominal distension, and a case of nausea. Besides, 5 RCTs (Shi and Liu, 2011; Fan and He, 2013; Gao and Wang, 2013; Song, 2018; Liu, 2020) associated with SF did not report ADRs or ADEs. Four RCTs (Long et al., 2000; Wu and Teng, 2001; Yu and Liu, 2004; Liu, 2007) mentioned ADRs of Sm, including dizziness (1 case in 1 study), head bilges (2 cases in 1 study), palpitation (3 cases in 2 studies), thirst, and mouth/tongue dryness (8 cases in 1 study). While two cases of mild facial flushing and dizziness were recorded in one study (Li, 2007) involving SM. Moreover, only one RCT (Ge et al., 2018) of XML observed two cases of dizziness. After rest and slowing down the infusion rate, these symptoms relieved, which did not affect the study.

### Cluster analysis

**FIGURE 6:**
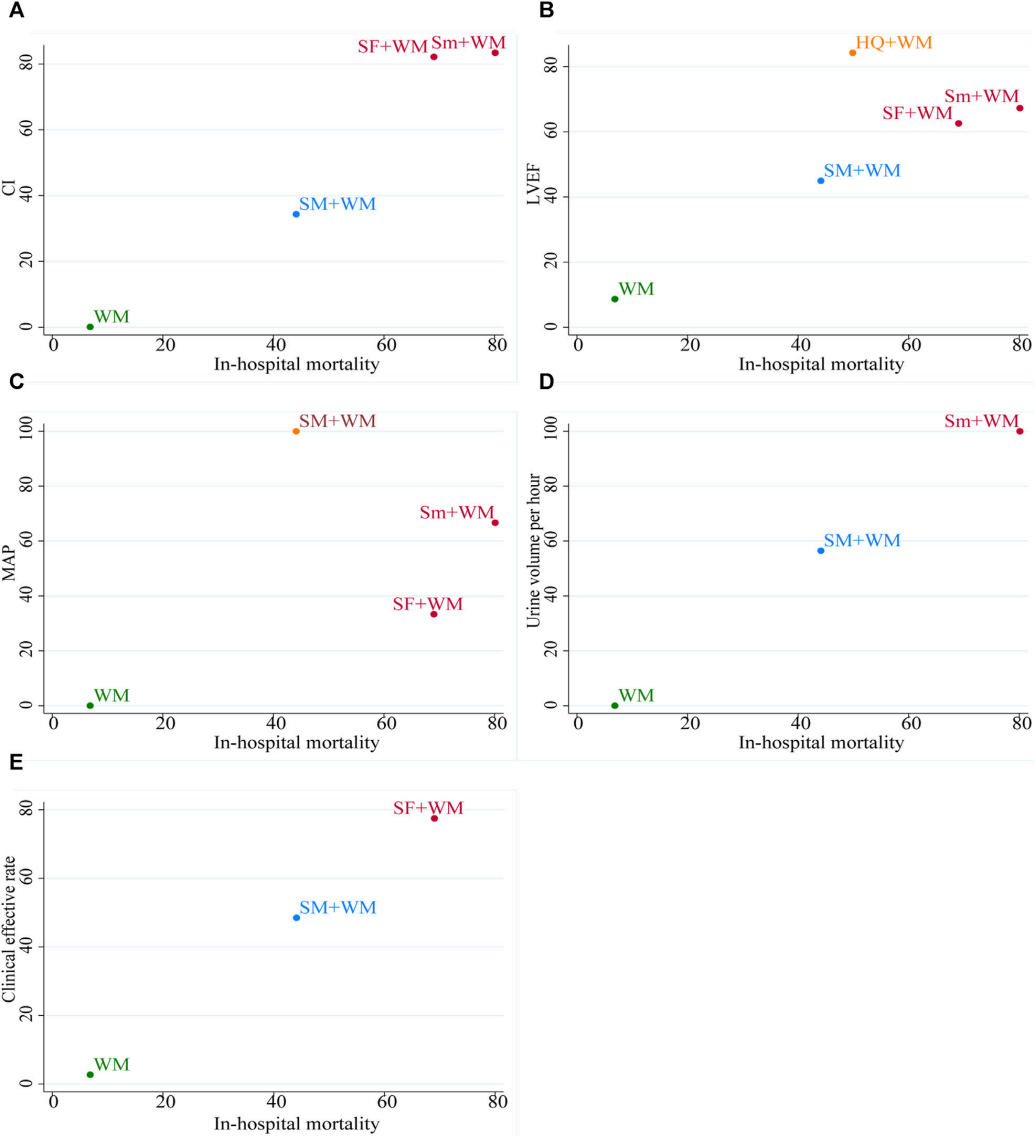
The plot of cluster analysis. It displays five outcomes, with interventions of the same color grouped together as a cluster. In the upper right corner, there are interventions that suggest optimal therapy for two distinct outcomes.

To comprehensively compare the efficacy of five interventions, we conducted a cluster analysis on six outcomes, including in-hospital mortality and five other outcomes. Figure 6 revealed that, with regard to the in-hospital mortality and the CI, the in-hospital mortality and the LVEF, and the in-hospital mortality and the MAP, Sm + WM and SF + WM exhibited more benefits. In relation to the in-hospital mortality and the hourly urine output, Sm + WM, located in the upper right corner, proved to be the primary treatment. SF + WM was found to be preferred in terms of in-hospital mortality and the clinical effective rate. However, in these five cluster analyses, WM was always situated in the lower left corner, which indicated that compared to the other three treatments, WM showed the worst therapeutic effect.

### Sensitivity analysis

We performed this analysis to further evaluate the robustness and reliability of in-hospital mortality. Two studies (Long et al., 2000; Ding and Xu, 2006) were excluded due to their small sample sizes, while the remaining eleven studies underwent a subsequent NMA. Notably, no significant discrepancies were observed when comparing the outcomes with those of the original NMA. A Bayesian ranking analysis was conducted, yielding the following order: Sm (84.04%), SF (67.26%), HQ (48.86%), SM (42.67%), and WM (7.18%). The sensitivity analysis further validated the robustness and reliability of the in-hospital mortality results.

### Funnel plot characteristics

**FIGURE 7:**
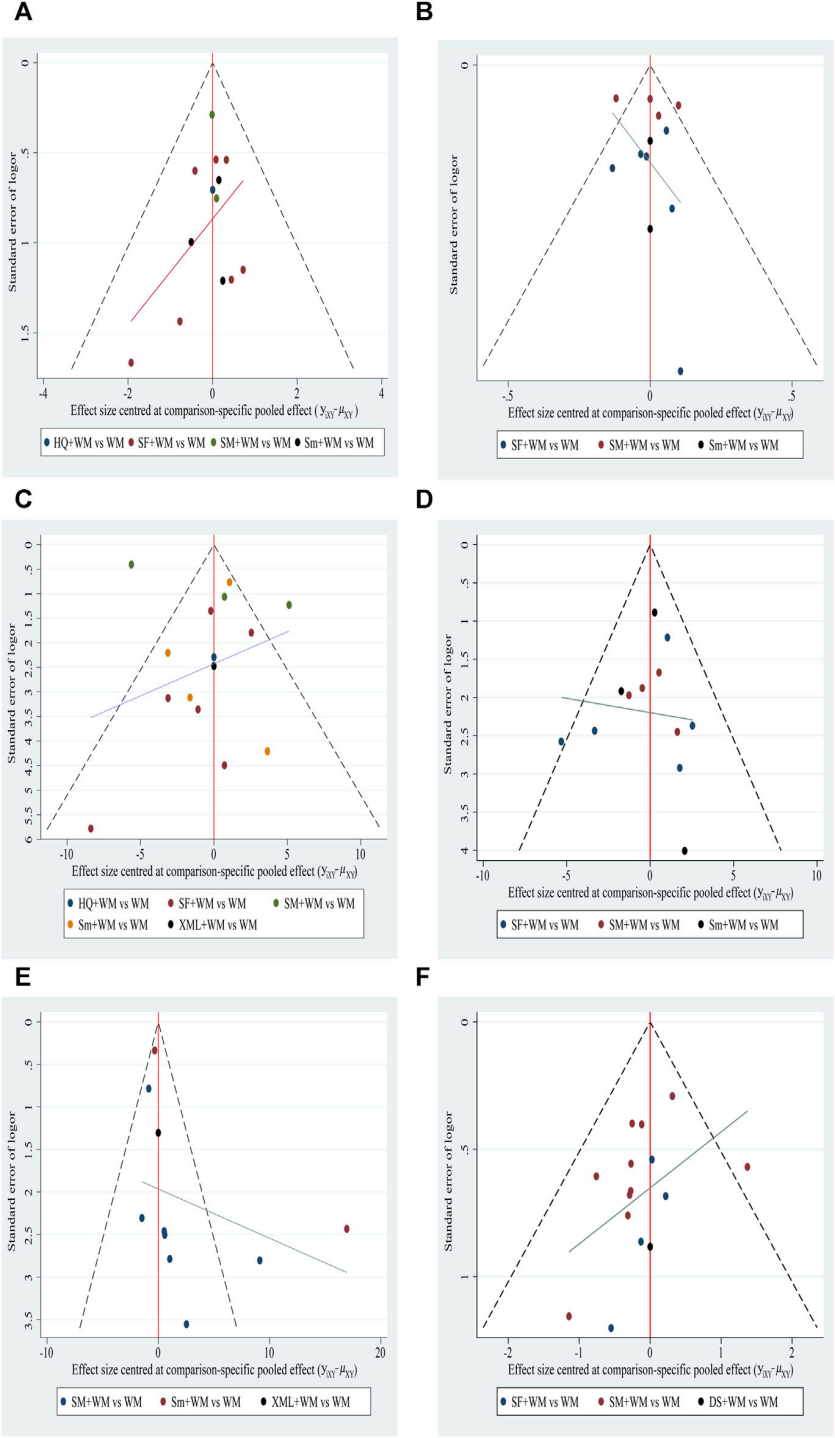
Funnel plot. **(A)** In-hospital mortality; **(B)** cardiac index (CI); **(C)** left ventricular ejection fraction (LVEF); **(D)** mean arterial pressure (MAP); **(E)** hourly urine output; **(F)** clinical effective rate.

There was one roughly symmetrical outcome, namely, CI, that indicated no publication bias. However, among other outcomes, the funnel plots showed asymmetry, suggesting a small sample size and publication bias. Details are shown in Figure 7.

## Discussion

This NMA included and evaluated 43 RCTs of CHIs in the treatment of CS, involving 2,665 subjects. Six different kinds of CHIs were analyzed, including SF, SM, Sm, DS, HQ, and XML. The nine typical outcomes that we selected were as follows: in-hospital mortality, CI, LVEF, MAP, hourly urine output, clinical effective rate, cTnI, CRP, and ADRs/ADEs. Initially, DIC and I^2^ values were calculated to determine the best-fitting model, and the heterogeneity of that model was estimated. The consistency assumption was not suitable for this NMA since there were non-closed rings.

According to the findings, Sm and SF performed better at lowering in-hospital mortality than WM alone. Sm ranked highest in reducing in-hospital mortality, improving CI, and increasing hourly urine output when combined with SUCRA values and placed second in LVEF and MAP. SF had the most remarkable impact on enhancing the clinical effective rate but came in second in CI. In terms of MAP, SM came out on top. Despite the fact that HQ was the most successful in raising LVEF, the efficacy of HQ needs more support from high-quality studies, considering that only one study was included in this research. Due to the small number of RCTs designed to analyze myocardial injury biomarkers and inflammatory factors, we could not conclude that CHIs + WM were superior to WM in reducing myocardial injury and inflammatory response. Furthermore, in terms of safety, approximately a third of the studies (14 RCTs) observed ADRs/ADEs involving SF, SM, Sm, and XML. And the ADRs/ADEs contained dizziness, palpitations, dry mouth and thirst, dry tongue, nausea, vomiting, facial flushing, skin itching, and facial flushing. The incidence of dizziness and palpitations was the highest. But given the small sample size, we were unable to draw the conclusion that CHIs + WM could considerably decrease ADRs/ADEs.

CS is a prevalent, intricate, refractory, and critical disease of low cardiac output that is relevant to various heart diseases and involves neuroendocrine system dysfunction, systemic immune inflammatory response, and microcirculation disorder (Editorial Board of Chinese Journal of Cardiology and Subspecialty Group of Acute and Intensive Cardiac Care of Chinese Society of Cardiology, 2019). This is the key reason for the high mortality and poor prognosis of CS. On the one hand, routine therapies, such as inotropic drugs, vasopressors, and IABP, have limited effects in reducing mortality; on the other hand, MCS devices like ECMO and percutaneous LVAD face drawbacks, including high cost and complicated operation (Goldberg et al., 2009; Zeymer et al., 2020). In clinical practice, the therapy of CHIs + WM displayed better performances compared to WM, including lowering in-hospital mortality, improving cardiac function, elevating hypertension, enhancing tissue perfusion, reducing ADRs/ADEs, and increasing clinical efficiency with lower costs.

CS belongs to the categories of “Jue syndrome,” “Tuo syndrome,” and “Jue Tuo syndrome” in traditional Chinese medicine (TCM). Similar clinical symptoms, such as “sudden convulsions, do not know the words,” “Jin Tuo, striae, sweating,” had been noted in “Huang Di Nei Jing” (Yang and Wang, 2021). Sm is made of effective *Panax ginseng* C.A.Mey and *Ophiopogon japonicus* (Thunb.) Ker Gawl extracts such as ginsenosides and Ophiopogon saponins. As per the TCM theory, *Panax ginseng* C.A.Mey tones vital qi and solidifies complex pulses, and *Ophiopogon japonicus* (Thunb.) Ker Gawl japonicus tones the lung and stomach, nourishes yin, and generates fluid. The combination of the two drugs plays a key role in benefiting qi, nourishing yin, and generating fluid. What is more, contemporary pharmacological research has substantiated the anti-inflammatory and cardioprotective properties of Sm (Yao et al., 2017; Tang et al., 2021; Cai et al., 2022). Through animal experiments, researchers further propose that the potential cardiovascular benefits of Sm can be attributed to its positive impact on cardiac performance, suppression of myocardial fibrosis, and mitigation of myocardial ischemia (Shi et al., 2019). Some researchers reveal that the anti-shock mechanism of Sm may be associated with its capacity to protect myocardial cells by reducing patient levels of myocardial injury markers (Shi et al., 2018). However, there are limited domestic and international studies on the mechanism of Sm. Therefore, we hope that more multi-center, large-sample studies can be verified. SM is mainly composed of ginsenosides, ophiopogonin, and Schisandrin B, which perform a vitally significant role in increasing coronary blood flow, protecting myocardial cells, and improving microcirculation (Cao et al., 2019). SF is made of *Panax ginseng* C.A.Mey and *Aconitum carmichaelii* Debeaux extracts, like ginsenosides and aconitine alkaloids. Modern research shows that SF may play an anti-shock role by improving hemodynamics, inhibiting the inflammatory response, and improving microcirculation (Jiang et al., 2017; Zhuo et al., 2018; Zhang et al., 2019; Liu et al., 2020). Considering the one study, more high-quality evidence is desired for the effectiveness of HQ. As for safety, studies indicate that the frequent use and rapid administration of CHIs are frequently attributed to the occurrence of side effects and ADRs/ADEs (Zhu, 2011). In the reported ADRs, allergic reactions are the most common (Li et al., 2013; Deng et al., 2023). In addition, due to the multiple targets of CHIs, there is a potential risk of drug interactions when used in combination with other medications (Zhang et al., 2017). However, under proper usage, the incidence of adverse reactions from traditional Chinese medicine injections is very low, and serious harm to the human body is extremely rare (Gao, 2017; Wang et al., 2021).

This study has the following advantages. Above all, as we know, it is the first NMA to comprehensively assess the efficacy and safety of diverse CHIs applied to treat patients with CS. And for clinicians, the findings can indicate a novel perspective on CHIs plus WM, especially Sm plus WM, in treating CS. More importantly, in this study, we not only glance at in-hospital mortality, cardiac function indicators, and the peripheral tissue perfusion indicator but also analyze ADRs/ADEs and inflammation biomarkers represented by CRP. In addition to reflecting the efficacy of CHIs, in-hospital mortality is also an excellent indicator of the societal disease burden. Inflammation biomarkers are strongly connected to both the CS’s pathophysiology and the mechanism of drug action. Hence, this study can offer a significant point of reference for healthcare professionals.

## Limitation

There are some limitations to this NMA. First of all, there was no clinical data from other nations because all the studies that made up this NMA were conducted in China with Chinese participants alone. Limited understanding and acceptance of TCM in other countries might be responsible for it. Second, the included studies were not of high caliber. Only 12 RCTs detailed the process of generating random sequences; 4 studies set up blinding; and none of the research endeavors furnished data concerning the concealment of allocation. This might be a consequence of the irregular writing or the lack of rigorous design in RCTs. The study quantity of certain CHIs is limited, potentially impacting comparisons and publication bias. In addition, the quality of the evidence body was negatively influenced by the lack of closed loops. This could be because it was challenging to directly evaluate the efficacy of various CHIs.

## Conclusion

In summary, CHIs + WM benefited patients with CS more significantly than WM alone. Among them, Sm + WM showed prominent performance in lowering in-hospital mortality, improving cardiac function, and restoring hourly urine output. As for SF + WM, it could significantly increase the clinical effective rate. SM + WM was proven to effectively improve the MAP. Through a comprehensive consideration of the outcomes, we believed that Sm + WM was the most suitable therapy for patients with CS. Given the limitations of this NMA, however, the conclusions aforementioned need to be further supported by larger sample sizes and multi-center studies. Furthermore, the safety monitoring of CHIs should be further strengthened.
