# Supplementary material for: Comparative efficacy of Chinese herbal injections in patients with cardiogenic shock (CS): a systematic review and Bayesian network meta-analysis of randomized controlled trials
Source: Front Pharmacol. 2024 Feb 27;15:1348360. doi: 10.3389/fphar.2024.1348360 (PMC10927829; doi:10.3389/fphar.2024.1348360)
Supplement: Supplementary file 1 [file Table1.docx]

**Supplement 1. Composition of the Chinese herbal injections**

Table S1. Composition of the Chinese herbal injections.

| Drug name | Botanical plant name | Family | Plant part used | Component ingredients to be measured | |
| --- | --- | --- | --- | --- | --- |
| Shenfu injection | *Panax ginseng* C.A.Mey.  *Aconitum carmichaeli* Debeaux | Araliaceae  Ranunculaceae | Root and rhizome  Root | Ginsenoside, aconitine | |
| Shengmai injection | *Panax ginseng* C.A.Mey.  *Ophiopogon japonicus* (Thunb.) Ker Gawl.  *Schisandra chinensis* (Turcz.) Baill. | Araliaceae  Asparagaceae  Schisandraceae | Root and rhizome  Root  Fruit | ginsenosides, ophiopogonin, and Schisandrin B | |
| Shenmai injection | *Panax ginseng* C.A.Mey.  *Ophiopogon japonicus* (Thunb.) Ker Gawl. | Araliaceae  Asparagaceae | Root and rhizome  Root | ginsenosides and Ophiopogon saponins |  |
| Danshen injection | *Salvia miltiorrhiza* Bunge | Lamiaceae | Root and rhizome | Danshen ketones and salvianolic acids. | |
| Huangqi injection | *Astragalus mongholicus* Bunge | Fabaceae | Root | Astragaloside and astragalus polysaccharide. | |
| Xinmailong injection | *Periplaneta americana* (Linnaeus) | Blattidae | - | Compound nucleotide bases and binding amino acids. | |
