# Supplementary material for: Comparative efficacy of Chinese herbal injections in patients with cardiogenic shock (CS): a systematic review and Bayesian network meta-analysis of randomized controlled trials
Source: Front Pharmacol. 2024 Feb 27;15:1348360. doi: 10.3389/fphar.2024.1348360 (PMC10927829; doi:10.3389/fphar.2024.1348360)
Supplement: Supplementary file 2 [file Table7.doc]

**Supplement 7. Certainty of the evidence**

Table S13. The level of evidence of the in-hospital mortality.

| Comparison | Number of studies | Within-study bias | Reporting bias | Indirectness | Imprecision | Heterogeneity | Incoherence | Confidence rating |
| --- | --- | --- | --- | --- | --- | --- | --- | --- |
| HQ+WM: WM | 1 | Some concerns | Some concerns | No concerns | Major concerns | No concerns | Major concerns | Very low |
| SF+WM: WM | 7 | Some concerns | Some concerns | No concerns | No concerns | No concerns | Major concerns | Very low |
| SM+WM: WM | 2 | Some concerns | Some concerns | No concerns | Some concerns | No concerns | Major concerns | Very low |
| Sm+WM: WM | 3 | Some concerns | Some concerns | No concerns | No concerns | Some concerns | Major concerns | Very low |
| HQ+WM: SF+WM | 0 | Some concerns | Some concerns | No concerns | Major concerns | No concerns | Major concerns | Very low |
| HQ+WM: SM+WM | 0 | Some concerns | Some concerns | No concerns | Major concerns | No concerns | Major concerns | Very low |
| HQ+WM: Sm+WM | 0 | Some concerns | Some concerns | No concerns | Major concerns | No concerns | Major concerns | Very low |
| SF+WM: SM+WM | 0 | Some concerns | Some concerns | No concerns | Major concerns | No concerns | Major concerns | Very low |
| SF+WM: Sm+WM | 0 | Some concerns | Some concerns | No concerns | Major concerns | No concerns | Major concerns | Very low |
| Sm+WM: SM+WM | 0 | Some concerns | Some concerns | No concerns | Major concerns | No concerns | Major concerns | Very low |

Abbreviations: SF: Shenfu injection; SM: Shengmai injection; HQ: Huangqi injection; WM: Western medicine.

Table S14. The level of evidence of the cardiac index (CI).

| Comparison | Number of studies | Within-study bias | Reporting bias | Indirectness | Imprecision | Heterogeneity | Incoherence | Confidence rating |
| --- | --- | --- | --- | --- | --- | --- | --- | --- |
| SF+WM: WM | 6 | Some concerns | Low risk | No concerns | No concerns | No concerns | Major concerns | Very low |
| SM+WM: WM | 4 | Some concerns | Low risk | No concerns | No concerns | Some concerns | Major concerns | Very low |
| Sm+WM: WM | 2 | Some concerns | Low risk | No concerns | No concerns | Some concerns | Major concerns | Very low |
| SF+WM: SM+WM | 0 | Some concerns | Low risk | No concerns | No concerns | Some concerns | Major concerns | Very low |
| SF+WM:Sm+WM | 0 | Some concerns | Low risk | No concerns | No concerns | Some concerns | Major concerns | Very low |
| Sm+WM: SM+WM | 0 | Some concerns | Low risk | No concerns | No concerns | Some concerns | Major concerns | Very low |

Abbreviations: SF: Shenfu injection; SM: Shengmai injection; Sm: Shenmai injection; WM: Western medicine.

Table S15. The level of evidence of the left ventricular ejection fraction (LVEF).

| Comparison | Number of studies | Within-study bias | Reporting bias | Indirectness | Imprecision | Heterogeneity | Incoherence | Confidence rating |
| --- | --- | --- | --- | --- | --- | --- | --- | --- |
| HQ+WM: WM | 1 | Some concerns | Some concerns | No concerns | Some concerns | No concerns | Major concerns | Very low |
| SF+WM: WM | 6 | Some concerns | Some concerns | No concerns | No concerns | Some concerns | Major concerns | Very low |
| SM+WM: WM | 3 | Some concerns | Some concerns | No concerns | No concerns | Some concerns | Major concerns | Very low |
| Sm+WM: WM | 4 | Some concerns | Some concerns | No concerns | No concerns | Some concerns | Major concerns | Very low |
| WM: XML+WM | 1 | Some concerns | Some concerns | No concerns | Some concerns | Some concerns | Major concerns | Very low |
| HQ+WM: SF+WM | 0 | Some concerns | Some concerns | No concerns | No concerns | Some concerns | Major concerns | Very low |
| HQ+WM: SM+WM | 0 | Some concerns | Some concerns | No concerns | Some concernS | Some concerns | Major concerns | Very low |
| HQ+WM: Sm+WM | 0 | Some concerns | Some concerns | No concerns | Some concerns | Some concerns | Major concerns | Very low |
| HQ+WM: XML+WM | 0 | Some concerns | Some concerns | No concerns | Some concerns | Some concerns | Major concerns | Very low |
| SF+WM: SM+WM | 0 | Some concerns | Some concerns | No concerns | No concerns | Major concerns | Major concerns | Very low |
| SF+WM: Sm+WM | 0 | Some concerns | Some concerns | No concerns | No concerns | Major concerns | Major concerns | Very low |
| SF+WM: XML+WM | 0 | Some concerns | Some concerns | No concerns | Some concerns | Some concerns | Major concerns | Very low |
| Sm+WM: SM+WM | 0 | Some concerns | Some concerns | No concerns | No concerns | Major concerns | Major concerns | Very low |
| SM+WM: XML+WM | 0 | Some concerns | Some concerns | Major concerns | Some concerns | No concerns | Major concerns | Very low |
| Sm+WM: XML+WM | 0 | Some concerns | Some concerns | No concerns | Some concerns | Some concerns | Major concerns | Very low |

Abbreviations: SF: Shenfu injection; SM: Shengmai injection; Sm: Shenmai injection; HQ: Huangqi injection; XML: Xinmailong injection; WM: Western medicine.

Table S16. The level of evidence of the mean arterial pressure (MAP).

| Comparison | Number of studies | Within-study bias | Reporting bias | Indirectness | Imprecision | Heterogeneity | Incoherence | Confidence rating |
| --- | --- | --- | --- | --- | --- | --- | --- | --- |
| SF+WM: WM | 5 | Some concerns | Some concerns | No concerns | No concerns | No concerns | Major concerns | Very low |
| SM+WM: WM | 4 | Some concerns | Some concerns | No concerns | No concerns | No concerns | Major concerns | Very low |
| Sm+WM: WM | 3 | Some concerns | Some concerns | No concerns | No concerns | No concerns | Major concerns | Very low |
| SF+WM: SM+WM | 0 | Some concerns | Some concerns | No concerns | No concerns | No concerns | Major concerns | Very low |
| SF+WM: Sm+WM | 0 | Some concerns | Some concerns | No concerns | No concerns | No concerns | Major concerns | Very low |
| Sm+WM: SM+WM | 0 | Some concerns | Some concerns | No concerns | No concerns | No concerns | Major concerns | Very low |

Abbreviations: SF: Shenfu injection; SM: Shengmai injection; Sm: Shenmai injection; WM: Western medicine.

Table S17. The level of evidence of the hourly urine output.

| Comparison | Number of studies | Within-study bias | Reporting bias | Indirectness | Imprecision | Heterogeneity | Incoherence | Confidence rating |
| --- | --- | --- | --- | --- | --- | --- | --- | --- |
| SM+WM: WM | 7 | Some concerns | Some concerns | No concerns | No concerns | Some concerns | Major concerns | Very low |
| Sm+WM: WM | 2 | Some concerns | Some concerns | No concerns | No concerns | No concerns | Major concerns | Very low |
| WM:XML+WM | 1 | Some concerns | Some concerns | No concerns | No concerns | Some concerns | Major concerns | Very low |
| Sm+WM: SM+WM | 0 | Some concerns | Some concerns | No concerns | No concerns | Some concerns | Major concerns | Very low |
| SM+WM:XML+WM | 0 | Some concerns | Some concerns | No concerns | No concerns | No concerns | Major concerns | Very low |
| Sm+WM: XML+WM | 0 | Some concerns | Some concerns | No concerns | No concerns | Some concerns | Major concerns | Very low |

Abbreviations: SM: Shengmai injection; Sm: Shenmai injection; XML: Xinmailong injection; WM: Western medicine.

Table S18. The level of evidence of the clinical effective rate.

| Comparison | Number of studies | Within-study bias | Reporting bias | Indirectness | Imprecision | Heterogeneity | Incoherence | Confidence rating |
| --- | --- | --- | --- | --- | --- | --- | --- | --- |
| DS+WM: WM | 1 | Some concerns | Some concerns | No concerns | Major concerns | No concerns | Major concerns | Very low |
| SF+WM: WM | 4 | Some concerns | Some concerns | No concerns | No concerns | No concerns | Major concerns | Very low |
| SM+WM: WM | 10 | Some concerns | Some concerns | No concerns | No concerns | No concerns | Major concerns | Very low |
| DS+WM: SF+WM | 0 | Some concerns | Some concerns | No concerns | Major concerns | No concerns | Major concerns | Very low |
| DS+WM:SM+WM | 0 | Some concerns | Some concerns | No concerns | Major concerns | No concerns | Major concerns | Very low |
| SF+WM: SM+WM | 0 | Some concerns | Some concerns | No concerns | Major concerns | No concerns | Major concerns | Very low |

Abbreviations: SF: Shenfu injection; SM: Shengmai injection; DS: Danshen injection; WM: Western medicine.
