# Supplementary material for: Comparative efficacy of Chinese herbal injections in patients with cardiogenic shock (CS): a systematic review and Bayesian network meta-analysis of randomized controlled trials
Source: Front Pharmacol. 2024 Feb 27;15:1348360. doi: 10.3389/fphar.2024.1348360 (PMC10927829; doi:10.3389/fphar.2024.1348360)
Supplement: Supplementary file 3 [file Table8.docx]

**Supplement 8. Surface under the cumulative ranking curve (SUCRA) results of the outcomes**

Table 2. Surface under the cumulative ranking curve (SUCRA) results of the outcomes.

|  | In-hospital mortality (%) | CI (%) | LVEF (%) | MAP (%) | Hourly urine output (%) | Clinical effective rate (%) |
| --- | --- | --- | --- | --- | --- | --- |
| WM | 6.86 | 0.1 | 8.65 | 0 | 0 | 2.73 |
| SF+WM | 69.03 | 82.16 | 62.6 | 33.33 | - | 77.46 |
| SM+WM | 44.12 | 34.35 | 44.97 | 100 | 56.47 | 48.48 |
| Sm+WM | 80.11 | 83.38 | 67.28 | 66.67 | 100 | - |
| DS+WM | - | - | - | - | - | 71.33 |
| HQ+WM | 49.88 | - | 84.25 | - | - | - |
| XML+WM | - | - | 32.25 | - | 43.53 | - |

Note: CI, Cardiac index; LVEF, Left ventricular ejection fraction; MAP, Mean arterial pressure; SF, Shenfu injection; SM, Shengmai injection; Sm, Shenmai injection; DS, Danshen injection; HQ, Huangqi injection; XML, Xinmailong injection; WM, Western medicine.
