# Supplementary material for: Comparative efficacy of Chinese herbal injections in patients with cardiogenic shock (CS): a systematic review and Bayesian network meta-analysis of randomized controlled trials
Source: Front Pharmacol. 2024 Feb 27;15:1348360. doi: 10.3389/fphar.2024.1348360 (PMC10927829; doi:10.3389/fphar.2024.1348360)
Supplement: Supplementary file 4 [file Table2.DOCX]

**Supplement 2. Extract and extraction process description of the Chinese herbal injections**

Table S2. Extract and extraction process description of the Chinese herbal injections.

| Injection | Extracts and Extraction Process Description |
| --- | --- |
| Shenfu injection | 1.5–18 parts of Panax ginseng C.A.Mey. extract and 1.5–18 parts of Aconitum carmichaelii Debeaux extract; mix the Panax ginseng C.A.Mey. extract and Aconitum carmichaelii Debeaux evenly; add 0.2% Tween-80; supplement with injection water to 1000 parts by volume; adjust the pH to 5-7 with sodium hydroxide; filter, fill, sterilize, and obtain the Shenfu injection. |
| Shengmai injection | Panax ginseng C.A.Mey. 100 g, Ophiopogon japonicus (Thunb.) Ker Gawl. 312 g, and Schisandra chinensis (Turcz.) Baill. 156 g were crushed into fine particles and extracted with ethanol for 5 times, 2 h each time. The extract was combined, refrigerated, and filtered, and the filtrate was concentrated into a thick paste. The injection water was added to 400 mL, stirred well, refrigerated, and filtered, and the filtrate was used for liquid preparation. Schisandra chinensis (Turcz.) Baill. collected 150 mL of distillate by steam distillation, refrigerated it, and used it for dispensing liquid. The dregs were decocted with water three times, for 40 minutes each time. The decoction was combined, filtered, and the filtrate concentrated into a thick paste. Ethanol was added for 2 times of alcohol precipitation, the first time to make the alcohol content up to 80%, the second time to make the alcohol content up to 85%, filtered, combined with the filtrate, recovered ethanol and concentrated to a thick paste, added water for injection to 200 mL, stirred, refrigerated, filtered, and the filtrate was boiled with an appropriate amount of activated carbon for 30 min, slightly cold, filtered to clear, for dispensing liquid. The preparation method of Ophiopogon japonicus (Thunb.) Ker Gawl. water solution is made into a clear Ophiopogon japonicus (Thunb.) Ker Gawl. aqueous solution of about 200 mL for liquid preparation. The water solution of Panax ginseng C.A.Mey., Schisandra chinensis (Turcz.) Baill. chinensis, Schisandra chinensis (Turcz.) Baill. chinensis, and Ophiopogon japonicus (Thunb.) Ker Gawl. were mixed and filtered. The filtrate was added with water for injection to 1 000 mL, and the pH of the solution was adjusted. |
| Shenmai injection | 100 g of *Panax ginseng* C.A.Mey. was crushed into fine particles and extracted with ethanol reflux for 5 times, 2 hours each time. The extract was combined, refrigerated, and filtered, and the filtrate was concentrated into a thick paste. Add water for injection to 400 mL, stir well, refrigerate, and filter. 312 g of *Ophiopogon japonicus* (Thunb.) Ker Gawl. was boiled with water three times, for 40 minutes each time. The decoction was combined, filtered, and the filtrate concentrated into a thick paste. Ethanol was added for two times of alcohol precipitation, and the alcohol content was 80% for the first time and 85% for the second time. After filtration, the filtrate was combined, and ethanol was recovered and concentrated into a thick paste. Add about 200 mL of water for injection, stir well, refrigerate, filter, add the appropriate amount of activated carbon to the filtrate, boil for 30 min, slightly cool, and filter until clear. The above *Panax ginseng* C.A.Mey. water solution and *Ophiopogon japonicus* (Thunb.) Ker Gawl. water solution were combined, mixed evenly, filtered, and the filtrate was added with water for injection to 1 000 mL. The pH value of the drug solution was adjusted to 7.5, filtered, sealed, and sterilized. |
| Danshen injection | 1500 g of Salvia miltiorrhiza Bunge was decocted three times with water: the first 2 hours, and the second and third 1.5 hours each. And the decoction was combined, filtered, and the filtrate concentrated to 750 mL under reduced pressure. Add ethanol to precipitate twice, using 75% ethanol for the first time and 85% ethanol for the second time, and filter after refrigeration each time. Then recover the ethanol from the filtrate and concentrate it to about 250 mL. After that, add injection water to 400 ml, mix well, and then refrigerate. Then filter, adjust the pH value to 6.8 with a 10% sodium hydroxide solution, and boil for half an hour. And filter again, add injection water to 1000 mL, fill in a container and seal, sterilize, and the Danshen injection was ready. |
| Huangqi injection | 2000 g of *Astragalus mongholicus* Bunge was decocted with water three times for 1.5 hours each time. The decoction was combined, filtered, and the filtrate concentrated to 1 mL, equivalent to 1 ~ 2g of the original medicinal material. Then the solution was precipitated with ethanol twice. The ethanol content in the first solution was 75% and 85% in the second, and it was refrigerated each time. Ethanol was recovered and concentrated to 1 mL, equivalent to 10g of the original medicinal material. Diluted with injection water to 1 mL, equivalent to 0.75 ~ 1g of the original medicinal material, and placed for 12 hours under refrigeration. Then filter and concentrate the filtrate to 1 mL, equivalent to 5 ~ 6g of the original medicinal material. Cool it down, and adjust the pH to 7.5 with a 20% sodium hydroxide solution. Boil the solution, add 0.125% activated carbon, and then boil again for 5 minutes. Filter it while it is hot, add injection water to 1000 ml, filter, and then adjust the pH to 7.5 with a 20% sodium hydroxide solution. Filter again, fill in a container and seal, sterilize, and the Huangqi injection was ready. |
| Xinmailong injection | Take dried bodies of Periplaneta americana (Linnaeus), extract with 90%–95% ethanol, and remove the oil to obtain a water-soluble extract. The active ingredients are adsorbed with activated carbon to obtain the extract. Then remove impurities such as pigments and allergens to obtain Periplaneta americana (Linnaeus) extract. Take 5g of Periplaneta americana (Linnaeus) extract, 20ml of polyethylene glycol-400, 0.9g of NaCl, add injection water to 100ml, and prepare Xinmailong injection. |
