# Supplementary material for: Comparative efficacy of Chinese herbal injections in patients with cardiogenic shock (CS): a systematic review and Bayesian network meta-analysis of randomized controlled trials
Source: Front Pharmacol. 2024 Feb 27;15:1348360. doi: 10.3389/fphar.2024.1348360 (PMC10927829; doi:10.3389/fphar.2024.1348360)
Supplement: Supplementary file 5 [file Table5.docx]

**Supplement 5. Search strategy**

Table S5. Search strategy used in PubMed.

| Number | Search terms | Results |
| --- | --- | --- |
| #1 | Cardiogenic shock [MeSH Terms] | 10926 |
| #2 | Cardiogenic shock [Title/Abstract] | 15638 |
| #3 | #1 OR #2 | 19225 |
| #4 | Chinese herbal injection [MeSH Terms] | 1230 |
| #5 | Chinese herbal injection [Title/Abstract] | 47 |
| #6 | Traditional Chinese medicine injection [Title/Abstract] | 121 |
| #7 | Traditional Chinese medicine [MeSH Terms] | 24296 |
| #8 | Traditional Chinese medicine [Title/Abstract] | 33077 |
| #9 | Huangqi [Title/Abstract] | 500 |
| #10 | Yiqifumai [Title/Abstract] | 37 |
| #11 | Shengmai [Title/Abstract] | 283 |
| #12 | Shenfu [Title/Abstract] | 306 |
| #13 | Shenmai [Title/Abstract] | 319 |
| #14 | Shenqifuzheng [Title/Abstract] | 19 |
| #15 | Danshen [Title/Abstract] | 1617 |
| #16 | Xinmailong [Title/Abstract] | 31 |
| #17 | Guanmaining [Title/Abstract] | 0 |
| #18 | #4 OR #5 OR #6 OR #7 OR #8 OR #9 OR #10 OR #11 OR #12 OR #13 OR #14 OR #15 OR #16 OR #17 | 49893 |
| #19 | randomized controlled trial [Publication Type] | 605170 |
| #20 | controlled clinical trial [Publication Type] | 695722 |
| #21 | random* [All Fields] | 1715790 |
| #22 | #19 OR #20 OR #21 | 1799659 |
| #23 | #3 AND #18 AND #22 | 6 |

Table S6. Search strategy used in Web of Science

| Number | Search terms | Results |
| --- | --- | --- |
| #1 | TOPIC: Cardiogenic shock | 28764 |
| #2 | TOPIC: Chinese herbal injection | 6056 |
| #3 | TOPIC: Traditional Chinese medicine injection | 4616 |
| #4 | TOPIC: Traditional Chinese medicine | 81847 |
| #5 | TOPIC: Huangqi | 564 |
| #6 | TOPIC: Yiqifumai | 36 |
| #7 | TOPIC: Shengmai | 316 |
| #8 | TOPIC: Shenqifuzheng | 20 |
| #9 | TOPIC: Shenfu | 517 |
| #10 | TOPIC: Shenmai | 370 |
| #11 | TOPIC: Danshen | 2388 |
| #12 | TOPIC: Xinmailong | 39 |
| #13 | TOPIC: guanmaining | 3 |
| #14 | #2 OR #3 OR #4 OR #5 OR #6 OR #7 OR #8 OR #9 OR #10 OR #11 OR #12 OR #13 | 86893 |
| #15 | #1 AND #14 | 22 |

Table S7. Search strategy used in Cochrane Library

| Number | Search terms | Results |
| --- | --- | --- |
| #1 | Cardiogenic shock: ti, ab, kw | 1614 |
| #2 | Chinese herbal injection: ti, ab, kw OR Traditional Chinese medicine injection: ti, ab, kw OR Traditional Chinese medicine: ti, ab, kw | 10883 |
| #3 | Huangqi: ti, ab, kw OR Yiqifumai: ti, ab, kw OR Shenfu: ti, ab, kw OR Shenmai: ti, ab, kw OR Shengmai: ti, ab, kw | 845 |
| #4 | Shenqifuzheng: ti, ab, kw OR Danshen: ti, ab, kw OR Xinmailong: ti, ab, kw OR Guanmaining: ti, ab, kw | 451 |
| #5 | #2 OR #3 OR #4 | 11853 |
| #6 | randomized controlled: ti,ab,kw | 985577 |
| #7 | #1 AND #5 AND #6 | 8 |

Table S8. Search strategy used in Embase

| Number | Search terms | Results |
| --- | --- | --- |
| #1 | 'cardiogenic shock': ti, ab, kw | 28716 |
| #2 | 'Chinese herbal injection': ti, ab, kw OR 'traditional Chinese medicine injection': ti, ab, kw OR 'traditional Chinese medicine': ti, ab, kw OR 'huangqi': ti, ab, kw OR 'shenfu': ti, ab, kw OR 'shenmai': ti, ab, kw OR 'shengmai': ti, ab, kw OR 'yiqifumai': ti, ab, kw OR 'shenqifuzheng': ti, ab, kw OR 'xinmailong': ti, ab, kw OR 'danshen': ti, ab, kw OR 'guanmaining': ti, ab, kw | 45792 |
| #3 | 'random': ti, ab, kw | 440078 |
| #4 | #1 AND #2 AND #3 | 1 |

Table S9. Search strategy used in China National Knowledge Infrastructure (CNKI)

| Number | Search terms | Results |
| --- | --- | --- |
| #1 | (TS='cardiogenic shock') AND (TS='injection' OR TS='extractive' OR TS='for injection' OR TS='huangqi' OR TS='shenfu' OR TS='shenmai' OR TS='shengmai' OR TS='yiqifuzheng' OR TS='shenqifuzheng' OR TS='Xinmailong OR TS='Danshen' OR TS='Guanmaining') | 545 |

Table S10. Search strategy used in Wanfang Data

| Number | Search terms | Results |
| --- | --- | --- |
| #1 | (TS='cardiogenic shock') AND (TS='injection' OR TS='extractive' OR TS='for injection' OR TS='huangqi' OR TS='shenfu' OR TS='shenmai' OR TS='shengmai' OR TS='yiqifuzheng' OR TS='shenqifuzheng' OR TS='Xinmailong OR TS='Danshen' OR TS='Guanmaining') | 436 |

Table S11. Search strategy used in the Chinese Scientific Journal Database (VIP)

| Number | Search terms | Results |
| --- | --- | --- |
| #1 | (ti, kw='cardiogenic shock') AND (ti, kw='injection' OR ti, kw='extractive' OR ti, kw='for injection' OR ti, kw='huangqi' OR ti, kw='shengmai' OR ti, kw='yiqifumai' OR ti, kw='shenqifuzheng' OR ti, kw='Shenfu' OR ti, kw='Shenmai' OR ti, kw='Xinmailong OR ti, kw='Danshen' OR ti, kw='Guanmaining') | 278 |

Table S12. Search strategy used in China Biology Medicine disc (CBM)

| Number | Search terms | Results |
| --- | --- | --- |
| #1 | (ti, kw='cardiogenic shock') AND (ti, kw='injection' OR ti, kw='extractive' OR ti, kw='for injection' OR ti, kw='Shenfu' OR ti, kw='Shenmai' OR ti, kw='Shengmai' OR ti, kw='Shenqifuzheng' OR ti, kw='Huangqi' OR ti, kw='Yiqifumai' OR ti, kw='Xinmailong OR ti, kw='Danshen' OR ti, kw='Guanmaining') | 326 |
