# Supplementary material for: Comparative efficacy of Chinese herbal injections in patients with cardiogenic shock (CS): a systematic review and Bayesian network meta-analysis of randomized controlled trials
Source: Front Pharmacol. 2024 Feb 27;15:1348360. doi: 10.3389/fphar.2024.1348360 (PMC10927829; doi:10.3389/fphar.2024.1348360)
Supplement: Supplementary file 6 [file Table3.DOCX]

**Supplement 3. More details about the product information of CHIs**

Table S3 Details about the product information of 6 CHIs

| Injection name | Source | Therapeutic claims in TCM | Indications | Adverse drug reactions | Quality control reported? (Y/N) | Chemical analysis reporter? (Y/N) |
| --- | --- | --- | --- | --- | --- | --- |
| Shenfu injection | Ya'an Sanjiu Pharmaceutical Co., Ltd. | Reviving yang for resuscitation, reinforcing qi and preventing exhaustion. | Jue syndrome, palpitations, dizziness, panting, stomach pain, diarrhea, and numbness. | Tachycardia, allergic reactions, rash, dizziness, headache, belching, tremor, difficulty breathing, nausea, visual abnormalities, abnormal liver function, urinary retention, etc. | Y.  Z20043116 issued by China Food and Drug Administration | N |
| Shengmai injection | Huarun Sanjiu (Ya'an) Pharmaceutical Co., Ltd.; Shanghai and Hutchison Pharmaceutical Co., Ltd.; Jilin Jian Yisheng Pharmaceutical Co., Ltd.; Jiangsu Suzhong Pharmaceutical Group Biopharmaceutical Co., Ltd.; China National Pharmaceutical Group Yibin Pharmaceutical Co., Ltd. | Nourishing qi and nourishing yin, strengthening the pulse, and securing detachment. | Palpitations, shortness of breath, cold limbs, sweating, cardiogenic shock. | Dizziness, headache, seizures, head swelling, flushing, rash, itching, difficulty breathing, palpitations, laryngeal edema, anaphylactic shock, fever, chills, local skin reactions, nausea, vomiting, abdominal distension, diarrhea, stomach discomfort, dry mouth, chest tightness, decreased blood pressure, discomfort in the heart area, etc. | Y.  Z32020234 issued by China Food and Drug Administration | N |
| Shenmai injection | Dali Pharmaceutical Co., Ltd.; Sichuan Shenghe Pharmaceutical Co., Ltd.; Zhengda Qingchunbao Pharmaceutical Co., Ltd.; Hebei Shenwei Pharmaceutical Co., Ltd. | Strengthen qi and secure detachment; nourish yin; and generate fluids. | Shock, coronary heart disease, viral myocarditis, and chronic pulmonary heart disease. | Flushing, rash, itching, difficulty breathing, shortness of breath, palpitations, cyanosis, laryngeal edema, anaphylactic shock, chills, shivering, fever, fatigue, chest tightness, sweating, syncope, cough, chest tightness, chest pain, dry mouth, dry tongue, nausea, vomiting, abdominal pain, diarrhea, bloating, abnormal liver function tests, dizziness, headache, numbness, mental tension, etc. | Y.  Z13020889 issued by China Food and Drug Administration | N |
| Danshen injection | Sichuan Shenghe pharmaceutical co., Ltd. | Activating blood circulation, removing blood stasis, dredging pulses, and nourishing the heart. | Used for coronary heart disease, chest tightness, and angina pectoris. | Skin flushing or pallor, rash, itching, chills, laryngeal edema, dyspnea, palpitations, cyanosis, decreased blood pressure, and even shock, sweating, local skin reactions, fever and even high fever, fatigue, body pain, edema, cough, throat discomfort, chest tightness, breath holding, breathing difficulties, chest tightness, arrhythmia, nausea, abdominal pain, bloating, dry mouth, dizziness, headache, etc. | Y.  Z51021303 issued by China Food and Drug Administration | N |
| Huangqi injection | Shenwei Pharmaceutical Group Co., Ltd. | Tonify qi and nourish the origin, support the upright, and expel the evil. | Viral myocarditis, heart failure, and hepatitis. | Drug fever, drug rash, and redness and swelling at the injection site, acute allergic reactions, laryngeal edema, difficulty breathing, asthma, chest tightness, occasional liver dysfunction, vomiting, diarrhea, occasional severe headache and renal dysfunction, etc. | N | N |
| Xinmailong injection | Yunnan Tengyao Pharmaceutical Co., Ltd. | Tonify qi, activate blood circulation, unblock yang, and promote water metabolism. | Used for chronic congestive heart failure, palpitations, edema, shortness of breath, dull complexion, and cyanosis of the lips. | Rash, itching, shortness of breath, difficulty breathing, chest tightness, palpitations, anaphylactic shock, chills, shivering, fever, flushing, sweating, redness, cyanosis, dizziness, headache, tremor, nausea, vomiting, abdominal distension, abdominal pain, etc. | N | N |

Table S4 More details about the product information

| Study ID | Injection name | Source | Species | Quality control reported? (Y/N) | Chemical analysis reporter? (Y/N) |
| --- | --- | --- | --- | --- | --- |
| Bi XF 2005 | Shenfu injection | Huarun Sanjiu (Ya'an) Pharmaceutical Co., Ltd. | *Panax ginseng* C.A.Mey. [Araliaceae]; *Aconitum carmichaelii* Debeaux [Ranunculaceae] | Y.  Z51020664 issued by China Food and Drug Administration | N |
| Ding L 2006 | Shengmai injection | Huarun Sanjiu (Ya'an) Pharmaceutical Co., Ltd. | *Panax ginseng* C.A.Mey. [Araliaceae]; O*phiopogon japonicus* (Thunb.) Ker Gawl. [Asparagaceae]; *Schisandra chinensis* (Turcz.) Baill. [Schisandraceae] | N | N |
| Du ZC 2018 | Shenfu injection | Huarun Sanjiu (Ya'an) Pharmaceutical Co., Ltd. | *Panax ginseng* C.A.Mey. [Araliaceae]; *Aconitum carmichaelii* Debeaux [Ranunculaceae] | N | N |
| Fan  WH 2013 | Shenfu injection | Huarun Sanjiu (Ya'an) Pharmaceutical Co., Ltd. | *Panax ginseng* C.A.Mey. [Araliaceae]; *Aconitum carmichaelii* Debeaux [Ranunculaceae] | N | N |
| Feng Y 2016 | Shengmai injection | Not mentioned | *Panax ginseng* C.A.Mey. [Araliaceae]; O*phiopogon japonicus* (Thunb.) Ker Gawl. [Asparagaceae]; *Schisandra chinensis* (Turcz.) Baill. [Schisandraceae] | N | N |
| Gao CB 2013 | Shenfu injection | Not mentioned | *Panax ginseng* C.A.Mey. [Araliaceae]; *Aconitum carmichaelii* Debeaux [Ranunculaceae] | N | N |
| Ge ZR 2018 | Xinmailong injection | Yunnan Tengyao Pharmaceutical Co., Ltd. | *Periplaneta americana* (Linnaeus) [Blattidae] | N | N |
| Jiang CX 2017 | Shengmai injection | Shanghai and Hutchison Pharmaceutical Co., Ltd. | *Panax ginseng* C.A.Mey. [Araliaceae]; O*phiopogon japonicus* (Thunb.) Ker Gawl. [Asparagaceae]; *Schisandra chinensis* (Turcz.) Baill. [Schisandraceae] | Y.  Z31020241 issued by China Food and Drug Administration | N |
| Jin YY2016 | Shenfu injection | Huarun Sanjiu (Ya'an) Pharmaceutical Co., Ltd. | *Panax ginseng* C.A.Mey. [Araliaceae]; *Aconitum carmichaelii* Debeaux [Ranunculaceae] | N | N |
| Lan CY 2014 | Shengmai injection | Not mentioned | *Panax ginseng* C.A.Mey. [Araliaceae]; O*phiopogon japonicus* (Thunb.) Ker Gawl. [Asparagaceae]; *Schisandra chinensis* (Turcz.) Baill. [Schisandraceae] | N | N |
| Li GY 2007 | Shengmai injection | Jilin Jian Yisheng Pharmaceutical Co., Ltd. | *Panax ginseng* C.A.Mey. [Araliaceae]; O*phiopogon japonicus* (Thunb.) Ker Gawl. [Asparagaceae]; *Schisandra chinensis* (Turcz.) Baill. [Schisandraceae] | N | N |
| Li SG 2016 | Shenfu injection | Huarun Sanjiu (Ya'an) Pharmaceutical Co., Ltd. | *Panax ginseng* C.A.Mey. [Araliaceae]; *Aconitum carmichaelii* Debeaux [Ranunculaceae] | Y.  Z20043116 issued by China Food and Drug Administration | N |
| Li WH 2016 | Shenmai injection | Dali Pharmaceutical Co., Ltd. | *Panax ginseng* C.A.Mey. [Araliaceae]; O*phiopogon japonicus* (Thunb.) Ker Gawl. [Asparagaceae] | N | N |
| Li YL 2012 | Shengmai injection | Huarun Sanjiu (Ya'an) Pharmaceutical Co., Ltd. | *Panax ginseng* C.A.Mey. [Araliaceae]; O*phiopogon japonicus* (Thunb.) Ker Gawl. [Asparagaceae]; *Schisandra chinensis* (Turcz.) Baill. [Schisandraceae] | N | N |
| Lin B 2020 | Shenfu injection | Not mentioned | *Panax ginseng* C.A.Mey. [Araliaceae]; *Aconitum carmichaelii* Debeaux [Ranunculaceae] | N | N |
| Liu DF 2015 | Shenmai injection | Not mentioned | *Panax ginseng* C.A.Mey. [Araliaceae]; O*phiopogon japonicus* (Thunb.) Ker Gawl. [Asparagaceae] | N | N |
| Liu DJ 2020 | Shenfu injection | Not mentioned | *Panax ginseng* C.A.Mey. [Araliaceae]; *Aconitum carmichaelii* Debeaux [Ranunculaceae] | N | N |
| Liu M 2007 | Shenmai injection | Sichuan Shenghe Pharmaceutical Co., Ltd. | *Panax ginseng* C.A.Mey. [Araliaceae]; O*phiopogon japonicus* (Thunb.) Ker Gawl. [Asparagaceae] | N | N |
| Long MZ 2000 | Shenmai injection | Zhengda Qingchunbao Pharmaceutical Co., Ltd. | *Panax ginseng* C.A.Mey. [Araliaceae]; O*phiopogon japonicus* (Thunb.) Ker Gawl. [Asparagaceae] | N | N |
| Mi ZY 2009 | Huangqi injection | Shenwei Pharmaceutical Group Co., Ltd. | *Astragalus mongholicus* Bunge [Fabaceae] | N | N |
| Pan W 2015 | Shenfu injection | Huarun Sanjiu (Ya'an) Pharmaceutical Co., Ltd. | *Panax ginseng* C.A.Mey. [Araliaceae]; *Aconitum carmichaelii* Debeaux [Ranunculaceae] | N | N |
| Ren LQ 2016 | Shenfu injection | Not mentioned | *Panax ginseng* C.A.Mey. [Araliaceae]; *Aconitum carmichaelii* Debeaux [Ranunculaceae] | N | N |
| Shi BZ 2018 | Shenmai injection | Hebei Shenwei Pharmaceutical Co., Ltd. | *Panax ginseng* C.A.Mey. [Araliaceae]; O*phiopogon japonicus* (Thunb.) Ker Gawl. [Asparagaceae] | Y.  Z13020889 issued by China Food and Drug Administration | N |
| Shi CZ 2011 | Shenfu injection | Not mentioned | *Panax ginseng* C.A.Mey. [Araliaceae]; *Aconitum carmichaelii* Debeaux [Ranunculaceae] | N | N |
| Song CJ 2018 | Shengmai injection | Huarun Sanjiu (Ya'an) Pharmaceutical Co., Ltd. | *Panax ginseng* C.A.Mey. [Araliaceae]; O*phiopogon japonicus* (Thunb.) Ker Gawl. [Asparagaceae]; *Schisandra chinensis* (Turcz.) Baill. [Schisandraceae] | Y.  Z32020234 issued by China Food and Drug Administration | N |
| Song SP 2022 | Shenfu injection | Huarun Sanjiu (Ya'an) Pharmaceutical Co., Ltd. | *Panax ginseng* C.A.Mey. [Araliaceae]; *Aconitum carmichaelii* Debeaux [Ranunculaceae] | Y.  Z51020664 issued by China Food and Drug Administration | N |
| Su BL 2020 | Shenfu injection | Huarun Sanjiu (Ya'an) Pharmaceutical Co., Ltd. | *Panax ginseng* C.A.Mey. [Araliaceae]; *Aconitum carmichaelii* Debeaux [Ranunculaceae] | Y.  Z51020664 issued by China Food and Drug Administration | N |
| Su YF 2021 | Shengmai injection | Not mentioned | *Panax ginseng* C.A.Mey. [Araliaceae]; O*phiopogon japonicus* (Thunb.) Ker Gawl. [Asparagaceae]; *Schisandra chinensis* (Turcz.) Baill. [Schisandraceae] | N | N |
| Wang PF 2003 | Shengmai injection | Jiangsu Suzhong Pharmaceutical Group Biopharmaceutical Co., Ltd. | *Panax ginseng* C.A.Mey. [Araliaceae]; O*phiopogon japonicus* (Thunb.) Ker Gawl. [Asparagaceae]; *Schisandra chinensis* (Turcz.) Baill. [Schisandraceae] | N | N |
| Wang QS 2011 | Shengmai injection | China National Pharmaceutical Group Yibin Pharmaceutical Co., Ltd. | *Panax ginseng* C.A.Mey. [Araliaceae]; O*phiopogon japonicus* (Thunb.) Ker Gawl. [Asparagaceae]; *Schisandra chinensis* (Turcz.) Baill. [Schisandraceae] | N | N |
| Wei ZC 2013 | Shengmai injection | Not mentioned | *Panax ginseng* C.A.Mey. [Araliaceae]; O*phiopogon japonicus* (Thunb.) Ker Gawl. [Asparagaceae]; *Schisandra chinensis* (Turcz.) Baill. [Schisandraceae] | N | N |
| Wu WS 2001 | Shenmai injection | Not mentioned | *Panax ginseng* C.A.Mey. [Araliaceae]; O*phiopogon japonicus* (Thunb.) Ker Gawl. [Asparagaceae] | N | N |
| Xiong WS 2009 | Shenfu injection | Not mentioned | *Panax ginseng* C.A.Mey. [Araliaceae]; *Aconitum carmichaelii* Debeaux [Ranunculaceae] | N | N |
| Xu WW 2014 | Shenfu injection | Huarun Sanjiu (Ya'an) Pharmaceutical Co., Ltd. | *Panax ginseng* C.A.Mey. [Araliaceae]; *Aconitum carmichaelii* Debeaux [Ranunculaceae] | N | N |
| Yang ZQ 2016 | Shengmai injection | Not mentioned | *Panax ginseng* C.A.Mey. [Araliaceae]; O*phiopogon japonicus* (Thunb.) Ker Gawl. [Asparagaceae]; *Schisandra chinensis* (Turcz.) Baill. [Schisandraceae] | N | N |
| Yu ZM 2004 | Shenmai injection | Hebei Shenwei Pharmaceutical Co., Ltd. | *Panax ginseng* C.A.Mey. [Araliaceae]; O*phiopogon japonicus* (Thunb.) Ker Gawl. [Asparagaceae] | N | N |
| Zhang HX 2019 | Shenfu injection | Huarun Sanjiu (Ya'an) Pharmaceutical Co., Ltd. | *Panax ginseng* C.A.Mey. [Araliaceae]; *Aconitum carmichaelii* Debeaux [Ranunculaceae] | Y.  Z20043116 issued by China Food and Drug Administration | N |
| Zhang J 2017 | Danshen injection | Sichuan Shenghe pharmaceutical co., Ltd. | *Salvia miltiorrhiza* Bunge [Lamiaceae] | Y.  Z51021303 issued by China Food and Drug Administration | N |
| Zhang R 2014 | Shenmai injection | Hebei Shenwei Pharmaceutical Co., Ltd. | *Panax ginseng* C.A.Mey. [Araliaceae]; O*phiopogon japonicus* (Thunb.) Ker Gawl. [Asparagaceae] | N | N |
| Zhang SQ 2012 | Shengmai injection | Not mentioned | *Panax ginseng* C.A.Mey. [Araliaceae]; O*phiopogon japonicus* (Thunb.) Ker Gawl. [Asparagaceae]; *Schisandra chinensis* (Turcz.) Baill. [Schisandraceae] | N | N |
| Zhang Y 2017 | Shenfu injection | Not mentioned | *Panax ginseng* C.A.Mey. [Araliaceae]; *Aconitum carmichaelii* Debeaux [Ranunculaceae] | N | N |
| Zhao JR 2009 | Shenfu injection | Not mentioned | *Panax ginseng* C.A.Mey. [Araliaceae]; *Aconitum carmichaelii* Debeaux [Ranunculaceae] | N | N |
| Zhao YB 2017 | Shenfu injection | Huarun Sanjiu (Ya'an) Pharmaceutical Co., Ltd. | *Panax ginseng* C.A.Mey. [Araliaceae]; *Aconitum carmichaelii* Debeaux [Ranunculaceae] | Y.  Z20043117 issued by China Food and Drug Administration | N |
