# Supplementary material for: Comparative efficacy of Chinese herbal injections in patients with cardiogenic shock (CS): a systematic review and Bayesian network meta-analysis of randomized controlled trials
Source: Front Pharmacol. 2024 Feb 27;15:1348360. doi: 10.3389/fphar.2024.1348360 (PMC10927829; doi:10.3389/fphar.2024.1348360)
Supplement: Supplementary file 8 [file Table6.docx]

**Supplement 6. Characteristics of the studies included in the network meta-analysis.**

Table 1. Characteristics of the studies included in the network meta-analysis.

| Study ID | N(E/C) | Sex(M/F) | Age(years) | Therapy of experiment group | Therapy of control group | Course(day) | Outcomes |
| --- | --- | --- | --- | --- | --- | --- | --- |
| Bi XF 2005 | 14/12 | 16/10 | 62.4±10.2 | Shenfu 40ml Qd + WM | WM | 14 | ③ |
| Ding L 2006 | 15/15 | 16/14 | 57.8±8.4 | Shengmai 30ml Q12h + WM | WM | 7 | ①②⑤ |
| Du ZC 2018 | 25/25 | 31/19 | E:40.21±7.77  C:41.33±7.56 | Shenfu 50ml Bid + WM | WM | 1 | ② |
| Fan  WH 2013 | 60/58 | 76/44 | E: 69.3±12.8  C:70.1±9.3 | Shenfu 40ml Qd + WM | WM | 14 | ①③⑨ |
| Feng Y 2016 | 45/45 | 67/23 | 43.4±7.1 | Shengmai 60ml Q12h + WM | WM | 7 | ②④ |
| Gao CB 2013 | 28/18 | 27/19 | E:65.3±18.4  C:65.1±17.7 | Shenfu 40ml Qd + WM | WM | 1 | ⑨ |
| Ge ZR 2018 | 50/50 | 74/26 | E:71.2±11.5  C:70.8±10.8 | Xinmailong 100~400mg Bid + WM | WM | 10 | ③⑤⑨ |
| Jiang CX 2017 | 30/30 | 39/21 | E: 70.71±6.00  C:69.43±8.25 | Shengmai 20ml Qd + WM | WM | 7 | ⑦ |
| Jin YY2016 | 30/30 | 52/8 | E:57±12  C:60±16 | ShenFu 100ml Qd + WM | WM | 3 | ①③⑦⑨ |
| Lan CY 2014 | 56/56 | 57/55 | E:8.4±11.7  C:59.6±10.8 | Shengmai 60ml Qd + WM | WM | 7 | ②④⑤⑥ |
| Li GY 2007 | 32/31 | 41/22 | E:53.2±10.7  C:51±9.8 | Shengmai 30ml Qd + WM | WM | 7~10 | ⑥⑨ |
| Li SG 2016 | 32/32 | 40/24 | 62.73±8.23 | Shenfu 100ml Qd + WM | WM | 14 | ① |
| Li WH 2016 | 40/40 | 40/40 | 62.5±5.4 | Shenmai 100ml Qd + WM | WM | 5 | ④⑤ |
| Li YL 2012 | 18/18 | 19/17 | E:56.3  C:57.2 | Shengmai 60ml Qd + WM | WM | 7 | ⑥ |
| Lin B 2020 | 36/36 | 40/32 | E:67.25±13.28  C:.85±11.36 | Shenfu 80ml Qd + WM | WM | 7 | ①②③④⑦ |
| Liu DF 2015 | 30/30 | 33/27 | E:44.2  C:44.8 | Shengmai 100ml Qd + WM | WM | 1 | ③ |
| Liu DJ 2020 | 10/10 | 13/7 | E:5±2.6  C:0.1±2.4 | Shenfu 100ml Qd + WM | WM | - | ④⑨ |
| Liu M 2007 | 17/17 | 19/15 | 57.4±17.2 | Shenmai 50ml Qd + WM | WM | 14 | ①③⑨ |
| Long MZ 2000 | 15/15 | 22/8 | 68.5±8.2 | Shenmai 40ml Qd + WM | WM | 14 | ①③⑨ |
| Mi ZY 2009 | 30/29 | 36/23 | E:62.33±10.27  C:60.43±11.55 | Huangqi 50ml Qd + WM | WM | 14 | ①③⑨ |
| Pan W 2015 | 30/30 | 31/29 | - | Shenfu 50ml Bid + WM | WM | 1 | ②⑥ |
| Ren LQ 2016 | 20/20 | 19/21 | E:61±1.2  C:59±3.9 | Shenfu 50ml Qd + WM | WM | 2 | ⑤ |
| Shi BZ 2018 | 56/56 | 87/25 | E:61.6±7.2  C:60.6±5.0 | Shenfu 200ml Qd + WM | WM | 5 | ②③④⑦⑨ |
| Shi CZ 2011 | 18/18 | 23/13 | E:71  C:70 | Shenfu 80ml Qd + WM | WM | 7 | ①③⑥⑨ |
| Song CJ 2018 | 60/60 | 80/40 | E:54.1±4.6  C:54.8±4.2 | Shenfu 40ml Qd + WM | WM | 7 | ②③⑥⑦⑨ |
| Song SP 2022 | 30/30 | 31/29 | E:74.91±11.50  C:77.22±10.84 | Shenfu 100ml Qd + WM | WM | 7 | ②④ |
| Su BL 2020 | 30/30 | 42/18 | E:63.87±10.18  C:64.33±9.70 | Shenfu 60ml Qd + WM | WM | 1 | ②④ |
| Su YF 2021 | 40/40 | 43/37 | E:64.75±13.0  C:64.19±15.4 | Shengmai 25~50ml Qd + WM | WM | - | ⑥ |
| Wang PF 2003 | 20/22 | 31/11 | E:59.40±16.27  C:58.11±17.27 | Shengmai 30~100ml Qd + WM | WM | 7 | ①⑥ |
| Wang QS 2011 | 25/25 | 35/15 | 46.5±7.2 | Shenmai 100ml Q12h + WM | WM | 7 | ④⑤⑥ |
| Wei ZC 2013 | 26/26 | 25/27 | 64.1±2.2 | Shengmai 100ml Bid + WM | WM | 7 | ④⑤⑥ |
| Wu WS 2001 | 34/34 | 30/38 | 65 | Shenmai 80ml Q Qd + WM | WM | 7 | ①⑨ |
| Xiong WS 2009 | 19/19 | 22/16 | 58.3±17.9 | Shenfu 60ml Qd + WM | WM | 14 | ①③ |
| Xu WW 2014 | 32/32 | 36/28 | E:72.23±15.12  C:71.47±16.28 | Shenfu 100ml Qd + WM | WM | 7~10 | ①②⑥ |
| Yang ZQ 2016 | 98/98 | 101/95 | E:57.03±6.74  C:56.27±40.31 | Shengmai 20~60ml Qd + WM | WM | 7 | ①③⑤⑥⑦ |
| Yu ZM 2004 | 32/30 | 39/23 | E:62.0±8.6  C:64.0±5.2 | Shenmai 20~50ml Qd + WM | WM | - | ⑨ |
| Zhang HX 2019 | 32/32 | 35/29 | E:69.4±9.2  C:68.5±9.0 | Shenfu 40ml Qd + WM | WM | 14 | ⑨ |
| Zhang J 2017 | 30/28 | 34/24 | 49.21±23.89 | Danshen 20ml Qd + WM | WM | 14 | ⑥ |
| Zhang R 2014 | 13/12 | 16/9 | 51.2± 9.1 | Shenmai 240~480ml Qd + WM | WM | 1~2 | ②③④ |
| Zhang SQ 2012 | 32/32 | 42/22 | 47.6±7.9 | Shengmai 100ml Bid + WM | WM | 7 | ④⑤⑥ |
| Zhang Y 2017 | 23/23 | 27/19 | 65.4±11.3 | Shenfu 100ml Qd + WM | WM | 3 | ④ |
| Zhao JR 2009 | 13/13 | 15/11 | E:59±2  C:58±4 | Shenfu 30ml Qd + WM | WM | 10~15 | ③⑥ |
| Zhao YB 2017 | 37/37 | 55/19 | E:6.31±4.24  C:7.29±4.27 | Shenfu 50ml Bid + WM | WM | 3 | ⑧ |

Note: ① In-hospital mortality; ② Cardiac index (CI); ③ Left ventricular ejection fraction (LVEF); ④ Mean arterial pressure (MAP); ⑤ Hourly urine output; ⑥ Clinical effective rate; ⑦ Level of cardiac troponin I (cTnI); ⑧ Level of c-reactive protein (CRP); ⑨ Adverse drug reactions (ADRs)/adverse drug events (ADEs). Abbreviations: Qd, quaque die (once a day); Bid, bis in die (twice a day); Q12h, quaque duodecim horas (every 12 hour).
